# Machine Learning based Analytical Framework for Automatic Hyperspectral Raman Analysis of Lithium-ion Battery Electrodes

**DOI:** 10.1038/s41598-019-54770-2

**Published:** 2019-12-03

**Authors:** Ankur Baliyan, Hideto Imai

**Affiliations:** NISSAN ARC, LTD., 1, Natsushima-cho, Yokosuka, Kanagawa 237-0061 Japan

**Keywords:** Characterization and analytical techniques, Raman spectroscopy, Characterization and analytical techniques, Batteries

## Abstract

The intelligence to synchronously identify multiple spectral signatures in a lithium-ion battery electrode (LIB) would facilitate the usage of analytical technique for inline quality control and product development. Here, we present an analytical framework (AF) to automatically identify the existing spectral signatures in the hyperspectral Raman dataset of LIB electrodes. The AF is entirely automated and requires fewer or almost no human assistance. The end-to-end pipeline of AF own the following features; (i) intelligently pre-processing the hyperspectral Raman dataset to eliminate the cosmic noise and baseline, (ii) extract all the reliable spectral signatures from the hyperspectral dataset and assign the class labels, (iii) training a neural network (NN) on to the precisely “labelled” spectral signature, and finally, examined the interoperability/reusability of already trained NN on to the newly measured dataset taken from the same LIB specimen or completely different LIB specimen for inline real-time analytics. Furthermore, we demonstrate that it is possible to quantitatively assess the capacity degradation of LIB via a capacity retention coefficient that can be calculated by comparing the LMO signatures extracted by the analytical framework (AF). The present approach is suited for real-time vibrational spectroscopy based industrial applications; multicomponent chemical reactions, chromatographic, spectroscopic mixtures, and environmental monitoring.

## Introduction

Raman spectroscopy is a fast, non-destructive, and inexpensive spectroscopic tool to probe the molecular variations for a given specimen. The characteristic fingerprinting pattern of Raman spectrum provides much-needed access to the sample information at the molecular level. Besides, extending the singleton Raman spectrum analysis to the hyper-spectral domain either spatially or temporally, considerably, will help vibrational spectroscopy to be put to use, routinely, for the quality control (QA/QC) and product development applications. At present, hyperspectral Raman spectroscopy is mostly confined to the laboratory-scale measurement and analysis, and there are specific reasons for it not being used as the real-time quality-control analytical tool; (i) the presence of the cosmic noise and background signature in the hyperspectral dataset, (ii) inability to accurately identify the number of spectral signature in the hyperspectral dataset, and (iii) non-transferability of the existing analytical model from one set of hyperspectral measurement data to another set of newly hyperspectral measured dataset^1^.

**Figure 1:**
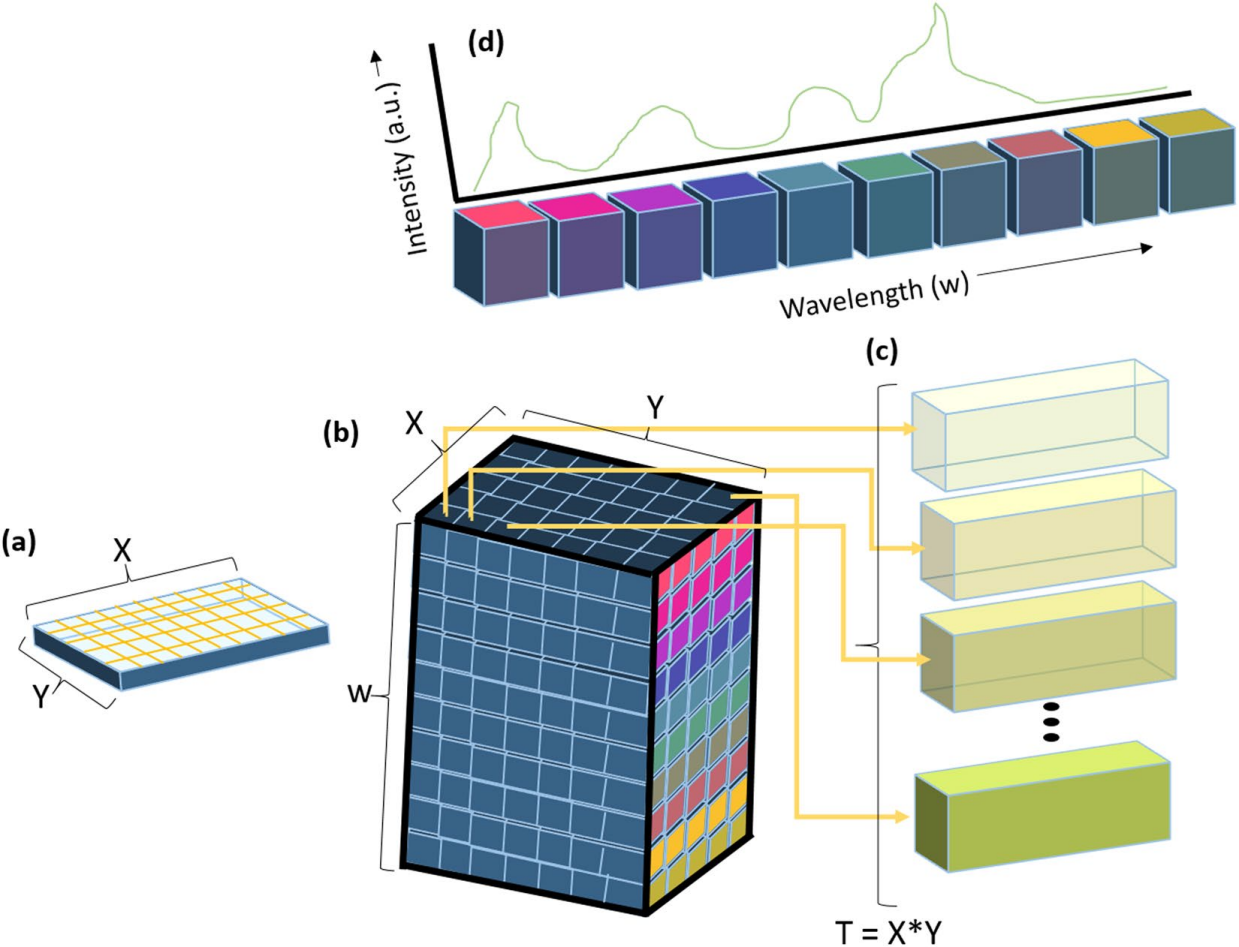
Illustration of hyperspectral images (3D data cubes), the spatial information is collected in the X–Y plane and the spectral information is represented in the Z-direction.

Hyperspectral Raman imaging caters the ability to image multiple chemical signatures simultaneously, and the spatial information is collected in the X–Y plane, and the spectral information is represented in the Z-direction, hyperspectral images are represented in the form of data cubes (Fig. 1). The analysis of hyperspectral Raman dataset (multidimensional) is usually cumbersome, complicated, and multistep process^2^. With modern scientific characterization instrumentation and faster than ever data generation capabilities, there is an urgent need for an innovative machine learning (ML) based computational framework to automate the entire life cycle of hyperspectral analysis^3^. The analytical model should be constructed with fewer or no human assistance. In addition, it is desired that the analytical model should be reusable, particularly on the newly measured dataset taken from the same specimen or completely different specimen^4^.

Recently, a number of ML techniques such as; principal component analysis (PCA), multiple curve resolution (MCR-ALS), independent component analysis (ICA), partial least square discriminant analysis (PLS-DA), voxel component analysis (VCA) and non-negative matrix factorization (NNF) have been applied for the identification/clustering of the significant spectral signatures from the test specimen^5–10^. Among all, MCR-ALS analysis, also referred to as un-mixing, has been extensively utilized to resolve the multiple pure spectral signatures and of their respective concentration components in the hyperspectral datasets. Essentially, MCR-ALS is responsive to the small variation within the specimen, and it helps to associates this variation to the contribution of the respective components (spectral/concentration) that can be measured and analysed, either qualitatively or quantitatively^3,11^. The indispensable condition for MCR-ALS analysis, to know beforehand, is the number of spectral signatures that are expected to be present in the hyperspectral datasets. An incorrect selection of the number of components can either lead to the inclusion of noise (i.e., overestimation) or loss of information (i.e., underestimation)^2,6^. In order to determine the appropriate number of components, to begin with, some methods such as; PCA, parallel analysis test, cross-validation, NMF-SO, and Kaiser criterion have been commonly used^5,6,12,13^. Unfortunately, for any chosen dataset processed via MCR-ALS, the end-results using such methods are not consistent and resulting in a different number of spectral and concentration profiles. This inconsistency curtails the reliability of the MCR-ALS analysis^2,6^.

The peculiar challenge with MCR-ALS is to determine the total number of components that exist in the chosen dataset; more importantly, the reliability of the emerged components, whether they are not falsely true. Although, Hiromi *et al*. have purposed an innovative approach by tagging the component “reliable” or “unreliable” based on the reproducibility of its appearance, regardless of the number of components chosen for analysis^6^. Nevertheless, the clustering of the concentration profiles, which is in unfolded spectral format, is quite vague and provides no visual confirmation to validate whether the rejected concentration profiles were really worth throwing out. Furthermore, the MCR-ALS analysis, encompass an analytical model that genuinely represents the characteristics of the test specimen, is a laborious process without having any reusability/transferability benefits, i.e., the newly established analytical model that is meant explicitly for a particular test specimen.

**Figure 2:**
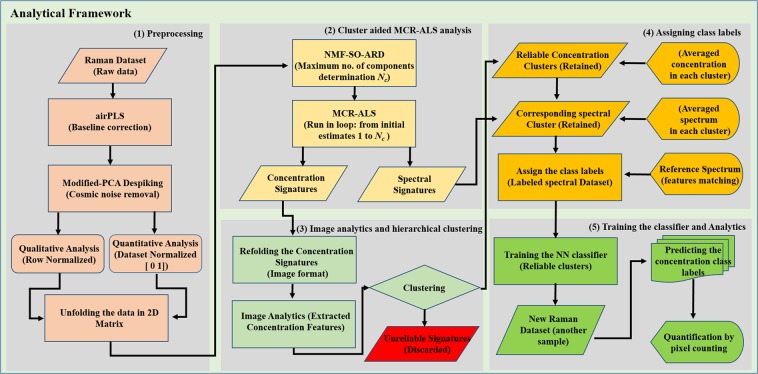
Machine learning based analytical framework (AF). The pipeline of the hyperspectral Raman analysis of LIB electrodes has domains; (i) intelligently pre-processing the hyperspectral Raman data with fewer or no human assistance, (ii) accurately identifying the reliable spectral signature from the hyperspectral dataset and assign the class labels, (iii) training a neural network (NN) on to the accurately “labelled” spectral signature, (iv) testing the reusability of already trained NN to evaluate other test samples in real-time.

Here, we propose an MCR-ALS based machine learning analytical framework (AF) to automate the entire pipeline of hyperspectral Raman analysis of lithium-ion battery (LIB) electrodes. The AF pipeline to make an analytical model involve; intelligently pre-processing the hyperspectral Raman data with fewer or no human assistance, accurately identifying the reliable spectral signature from the hyperspectral dataset and assign the class labels, training a neural network (NN) on to the accurately “labelled” spectral signature, and finally, testing the reusability of already trained NN to evaluate other test samples in real-time (Fig. 2 for schematic diagram). We started with data pre-processing; airPLS and modified-PCA based algorithms to remove the background and cosmic noise from the raw dataset. Subsequently, determine the appropriate number of components (*N*_*c*_) with NMF automatic relevance determination (NMF-SO-ARD) and performed the cluster-aided MCR-ALS analysis by sequentially changing the number of expected component from n = 1 to *N*_*c*_, and tagging the component “reliable” or “unreliable” based on the reproducibility of its appearance (see the image 2).

The reliable component periodically appears irrespective of the number of components chosen n = 1, 2, 3… *N*_*c*_. To cluster the reliable components, the concentration profiles were refolded to form images because, in contrast to the spectral format, the concentration profile provides better visual representation in pictographic format. Clustering of all the refolded concentration image dataset classifies concentration images into distinct clusters (collection of similar images). Clusters containing higher co-relation and reproducible images were stamped, as reliable clusters, and was retained for *NN* modelling. Each retained cluster was marked as a known class label. However, clusters having dissimilar images were rejected. Noting, by averaging all the concentration images and corresponding spectral profiles, within an individual retained cluster, provides a trusted singleton concentration image and spectrum profile.

Lastly, we trained a neural network (TNN) using the retained spectral data set and spectrum class labels and accessed the accuracy of TNN against the same dataset that the TNN was derived. In addition, examined the interoperability of the TNN from one dataset to another dataset, extending the benefit of reusable TNN analytical model. Such a trained model is very efficient and reliable, considering the analysis can be completed in real-time. The present approach is suited for real-time vibrational spectroscopy based quality control (QA/QC) and product development tools for routine industrial applications such as; multicomponent chemical reactions, industrial processes, chromatographic, spectroscopic mixtures, and environmental monitoring, etc.

## Necessity of the Neural Network Model

It is essential to illustrate what sorts of benefits will be served by the neural network model (NN). The limitations of routine MCR-ALS analysis are that it consumes considerable time before reaching an optimal solution of a given data-set, i.e., concentration maps and respective spectrum. Usually, the background subtraction, cosmic noise, MCR-ALS, and clustering required roughly forty to fifty minutes (on a personal computer- see details in the results section). In addition, the instrument-operator has no idea of the quality of the acquisition data being recorded during the characterization of the substrate. Considering, the analytics is often done in offline-mode that need separate plugins for background subtraction/cosmic noise/MCR-ALS. In a nutshell, MCR-ALS based analytical model is exclusive to the data-set it was created and cannot be reused to evaluate other similar specimens from the same batch or different specimen from the other batches, i.e., restricting the reusability/transferability of the MCR-ALS analytical model.

The central idea of training a NN is to do the analytics in nearly real-time, for the purpose of curtailing the analytics turnaround time to a few seconds after the acquisition of the data to pre-processing to spatially mapping the concentration profiles. In an ideal scenario, it would be interesting if one could build an analytical model using MCR-ALS via any chosen specimen dataset. Afterword, MCR-ALS based analytical model is translated to create an NN model. Such a strategy will not only assist the instrument-operator to judge the quality of the acquisition data instantly but at the same time, the neural network-based model can be used to evaluate other analogous samples in real-time that would save time, cost and energy. Therefore, the necessity of NN becomes imminent, and the use of NN allows hyper-spectral Raman analytics even with the personal computer almost in real-time.

## Material and Method

Lithium-ion battery (LIB) cells were procured (Panasonic, Japan) and subsequently analyzed by Raman spectroscopy (Alpha-300 confocal Raman microscope - WITec, GmbH) for hyperspectral Raman dataset. A typical cylindrical 18650-type LIB cell (2.1 Ah) consists of a graphite anode, Li(Ni_1−x−y_Mn_x_Co_y_)O_2_ (NMC) cathode and electrolyte; lithium hexafluorophosphate salt (LiPF_6_) dissolved in a mixture solvent of ethylene carbonate (EC), propylene carbonate (PC), ethyl methyl carbonate (EMC) and dimethyl carbonate (DMC). The diameter and height of LIB cell were 16 and 65 mm, respectively. The double-coating electrode was wound in the cylindrical cell; the thickness of single-side of the cathode was about 80 μm. Charge/discharge of LIB cells was done at 25 °C with 1C rate up to 500 cycles in the voltage range of 2.6–4.2 V. The battery cells were charged and discharged in constant current-constant voltage (CC-CV) and CC mode, respectively.

### Raman sample preparation and image acquisition

In total, three LIB cells were prepared, (a) Pristine sample – without any charge/discharge, (b) after 500 cycles of charge/discharge – from interior region of the cathode, and (c) after 500 cycles of charge/discharge – from outer region of the cathode (Fig. S1 for illustration). Here onward, (a), (b), and (c) will be addressed as pristine, 500_IN, and 500_Out samples, respectively. LIB cells were disassembled under argon (Ar) atmosphere inside an argon glove box. All the samples were rinsed in pure DMC and dried under vacuum, subsequently cross polished for the Raman characterization. Samples were sealed under argon in a specially designed sample holder to avoid air exposure. Raman spectra in the spectral range (100–3700 cm^−1^) were acquired with an Alpha-300 confocal Raman microscope (WITec, GmbH) using a solid-state 532 nm laser (laser power: 0.5 mW, optical lens: 40x, integration time: 1 second). Typically, for each sample thousands of spectra are acquired [pristine (scan width: 45 μm, Scan Height: 45 μm, Point per Line: 60, Lines per Image: 60), 500_IN (scan width: 55 μm, Scan Height: 20 μm, Point per Line: 72, Lines per Image: 26) and 500_Out samples (scan width: 45 μm, Scan Height: 45 μm, Point per Line: 60, Lines per Image: 60)], each containing position resolved information (Table T1 in SI). No, any additional data smoothing was done, and as measured data is used for further ML analysis.

### Analytical framework

Our analytical framework is built on the Matlab platform. At first, Raman imaging Dataset for each sample was exported into a.txt file and was converted to.mat file for further data processing. Extracting the vital information needs pre-processing of Raman spectra, i.e., background subtraction and despiking, followed by multivariate data analysis methods to generate the chemical composition and spectral signature. The main building blocks of the analytical framework is as follows; (1) Pre-processing the Raman dataset to remove the baseline and cosmic noise from the dataset, (2) Estimating the appropriate *N*_*c*_ with NMF-SO-ARD followed by cluster assisted-MCR-ALS regression analysis to extract the spectral and concentration profile by fitting the various number of components n = 1, 2, 3 …. *N*_*c*_, (3) Refolding the concentration profiles into image format, (4) feature extraction via image analytics and classification to assign the class labels, (5) designing a NN analytical model by training a NN on to the spectral profile with known class labels, and (6) reusing the already trained NN to test the random LIB Raman dataset for predicting the unknown concentration profile.

### Raman data-set

We collected 3600, 1872 & 3600 Raman spectra from pristine, 500_IN, and 500_Out LIB samples, respectively. A typical hyperspectral Raman image is a 3D dataset, hereafter addressed as a 3D spectral hypercube (Fig. 1), the structure of the 3D spectral hypercube X (*m* by *n* by *k*), where *m* and *n* axes represent spatial information of Raman image, and *k* number of data points per spectrum along the wavelength axis, respectively. Finally, 3D spectral hypercube X was folded into a 2D matrix by systematically placing recorded spectrum one over the other along the wavelength axis, transforming the hypercube (X: *l* × *k*), where *l* = m_*_n represent the total number of Raman measurement for a particular sample.

### Baseline correction & despiking

An adaptive iteratively reweighted penalized least-squares (airPLS) algorithm was used to remove the baseline from the Raman dataset (X: *l* × *k*), addressing the dataset as baseline-corrected dataset (X_B_: *l* × *k*) (see SI for Baseline Correction). In order to remove the cosmic noise from the baseline-corrected Raman dataset (X_B_: *l* × *k*), we have used the modified version of the PCA-despiking algorithm originally purposed by X. Zhang *et al*.^14^. In modified PCA-despiking algorithm concept is shown in Fig. S2, the baseline-corrected Raman dataset (X_B_: *l*,*k*) is sent as the input variable to the algorithm function, and the algorithm returned the despiked dataset as output variable (X_BD_: *l*,*k*), the despiked dataset is transposed (X_BD_: *k*,*l*), and again sent as an input (X_BD_: *k*,*l*) to the PCA-despiking algorithm function, subsequently, transposing the output variable (X_BD_: *k*,*l*) to make sure it returned to the initial dimension (X_BD_: *l*,*k*), this process is repeated until all the cosmic noise peaks were removed. Spike range and PCA variance cut-off were set to 20 and 0.85, irrespective of the dataset processed. (Code for the algorithm will be available after the publication of this article in supplementary information). The baseline-corrected dataset was normalized (row normalization) before the cluster-assisted MCR-ALS analysis.

### Cluster-assisted MCR-ALS

MCR-ALS analysis helps to identify the spectral (*S*^*t*^) and concentration profile (*C*) from the large Raman dataset. However, accuracy is limited by the fact that the spectral and concentration profile are not consistent across the number of components (n = 1, 2, 3 …) estimated by MCR-ALS analysis. In conventional MCR-ALS analysis, the spectrum data set is analyzed by specifying the desired number of components (it may be any positive integer)^15^. Whereas, in the cluster-aided-MCR-ALS (C-MCR-ALS), the MCR-ALS calculation was performed repeatedly by changing the number of components sequentially from one to an appropriate number of components (*N*_*c*_); *N*_*c*_, a positive integer, was estimated with NMF-SO-ARD was found to be 8, irrespective of any LIB sample^6^. A flow chart illustrating the process of cluster-aided C-MCR-ALS is shown in Fig. S3. Here, C-MCR-ALS analysis was performed for all three LIB samples, sequentially changing *n* from one to *N*_*c*_ = 8. For individual LIB sample, the total number of resulting components (*Z*) was 36 (concentration profile: *C* = 36 & spectral profile: *S*^*t*^ = 36). For a particular LIB sample, all the concentration profiles (*C*, *l* × *Z*) were combined into one dataset, and spectral profile (*S*^*t*^, *Z* × *k*) was put in another dataset. All concentration profiles (*C*, *l* × *Z*) were refolded back to form the Raman concentration images (*C*: m × n, *Z*) (RCI) dataset.

### Cluster Analysis and label assignment strategy

The strategy for the assignment of labels is as follows, we have the Raman concentration images (*C*: m × n, *Z*) (RCI) dataset and corresponding spectral profile (*S*^*t*^, *Z* × *k*) and there is a one-to-one mapping between the former and latter. If we are able to assign the class labels either of the two (RCIs or spectral profiles), others will automatically get the same class label. For training NN, labeled spectral profiles are needed, and that can be done simply by clustering of the spectral profiles. However, the challenge with clustering of the spectral profiles is that spectral clustering is quite vague, and visual confirmation of clusters is difficult given the large number of Raman feature space (wavenumber). Since the RCIs are in image format, clustering of RCIs provide the opportunity to validate the clusters visually and discarding of not trustworthy cluster becomes effortless. For this reason, the RCIs data-set was used for the clustering of RCIs into various clusters and based on cluster-IDs of each cluster the RCIs were assigned class labels. Because of the one-to-one relationship between the RCIs and spectral profiles, the spectral profiles also have access to the class labels. Having spectral profiles that are labeled, a neural network is ready to be trained.

### Cluster analysis and label assignment

Cluster analysis was performed on Raman concentration images (*C*: m × n, *Z*) (RCI) dataset using the Orange software with an add-on package of image analytics^16^. The image embedding widget uses the inception algorithm to transform each image from the RCI dataset into 2048 feature vectors, and hereafter the processed RCI dataset is addressed as an embedded dataset (*E*: 2048, *Z*). A correlation coefficient (CC), i.e., threshold, was chosen to classify the embedded dataset (E: 2048, Z) of similar features into various clusters using the hierarchical clustering widget. Hierarchical clustering segregates the RCI images-dataset into numerous clusters (*N*_*H*_: number of clusters), simultaneously, a numerical cluster-ID (*ID* = 1, 2, 3.. *N*_*H*_) is get assigned to each cluster. In order to bring clarity about what a cluster contained, a typical cluster has a bunch of images, from RCI images-dataset, whose feathers are similar to each other. Therefore, all the images in a typical cluster will have common cluster-ID (*ID*) automatically assigned. Since there is a one-to-one mapping of RCI image data-set (*C*: m × n, *Z*) and the images in clusters, by default, every RCI image and its corresponding spectral profile will also get the respective cluster-ID as the class label.

The clusters with higher correlation coefficient and minimum leaf size > = 3 were marked as ‘*reliable clusters*.’ Conversely, the clusters with lower correlation coefficient and minimum leaf size <3 were considered ‘*unreliable clusters’*. The reliable clusters were retained, whereas the unreliable clusters were not retained and discarded. Since there is a one-to-one mapping of RCI image data-set (*C*: m × n, *Z*) and the images in clusters, the images contained in unreliable clusters were also dropped from RCI (*C*: m × n, *Z*) dataset. Thereby, retained concentration profile (R_C: m × n, *Z*_*T*_) and corresponding spectral profile was moved to the new database (R_S^t^, *Z*_*T*_ × *k*); i.e., *Z* > *Z*_*T*_ = reliable number of concentration/spectral profile. Finally, averaging the concentration profiles and corresponding spectral signatures of each cluster provides a trusted singleton Raman concentration profile and spectrum, respectively. All of the reliable singleton Raman spectra were searched in Database (DB), and cluster-ID was replaced with the labels found in the DB; such as: carbon, LMO, background etc. However, if the cluster-ID was not found in the database it can be manually assigned such as ‘A’, ‘B’, ‘C’, and so on.

### Training the neural network from labeled class members

#### Configuration of the NN

A typical NN architecture (Fig. S4) is consists of four layers; one input layer, two hidden layers, and one output layer, respectively^17^. The input layer, sometimes, so-called “the visible layer,” connects the input variables (R_S^t^, *Z*_*T*_ × *k*) to the first hidden layer, where *Z*_*T*_ = reliable number of spectral profiles and *k* = number of data points per spectrum along the wavelength axis, respectively. In our network, first and second hidden layers have 10 and six perceptrons, respectively. The last layer in the NN is the output layer, received input from the last hidden layer of the network, and has the output nodes equal to the number of cumulative reliable clusters identified by C-MCR-ALS analysis^6^. Therefore, once the NN is trained, the output layer can predict the equal number of ‘class labels’. For instance, if the cumulative reliable clusters identified by C-MCR-ALS analysis is found to be ‘five,’ then the NN architecture needs ‘five output nodes, which in turn will predict five types of class labels. However, we found that the cumulative reliable clusters identified by C-MCR-ALS analysis, irrespective of the LIB Raman dataset (*Pristine*/*500_IN/500_Out*), was found to be four. Thereby, the NN architecture for all LIB Raman dataset (*NN*__*Pristine*_/*NN*__500_IN_/*NN*__500_Out_) had four output nodes in the last layer, irrespective of LIB Raman dataset.

#### Training the neural network

A neural network classifier was trained on to the reliable components (R_S^t^), extracted with C-MCR-ALS analysis (*Z*_*T*_: training-set), using the spectrum class labels from a particular LIB sample. Neural network (NN) corresponding to the each LIB Raman dataset (*Pristine/500_IN*/*500_Out*) was trained and named *NN*__ *Pristine*_, *NN*__500_IN_, and *NN*__500_Out_, respectively. The accuracy of each *NN* was examined with fivefold cross-validation.

#### Testing the neural network

Once the *NN* is trained, prediction of the class labels either from the same Raman dataset that the *NN* was derived or from the entirely new Raman dataset acquired using other LIB samples becomes straightforward. However, the test-data set should have undergone the baseline and cosmic-noise removal process (see baseline correction model section in SI). Three neural networks *NN*__ *Pristine*_, *NN*__500_IN_, and *NN*__500_Out_, was tested against the each LIB Raman dataset (*Pristine*/*500_IN*/*500_Out*) and the corresponding predicted concentration profiles as the class labels are plotted in image format. Finally, the efficacy of the purposed scheme is compared by cross-validating the results from the univariate (human intelligence), cluster-assisted MCR-ALS (Unsupervised intelligence), and Neural network predicted spectral classes (Supervised intelligence).

## Results and Discussion

**Figure 3:**
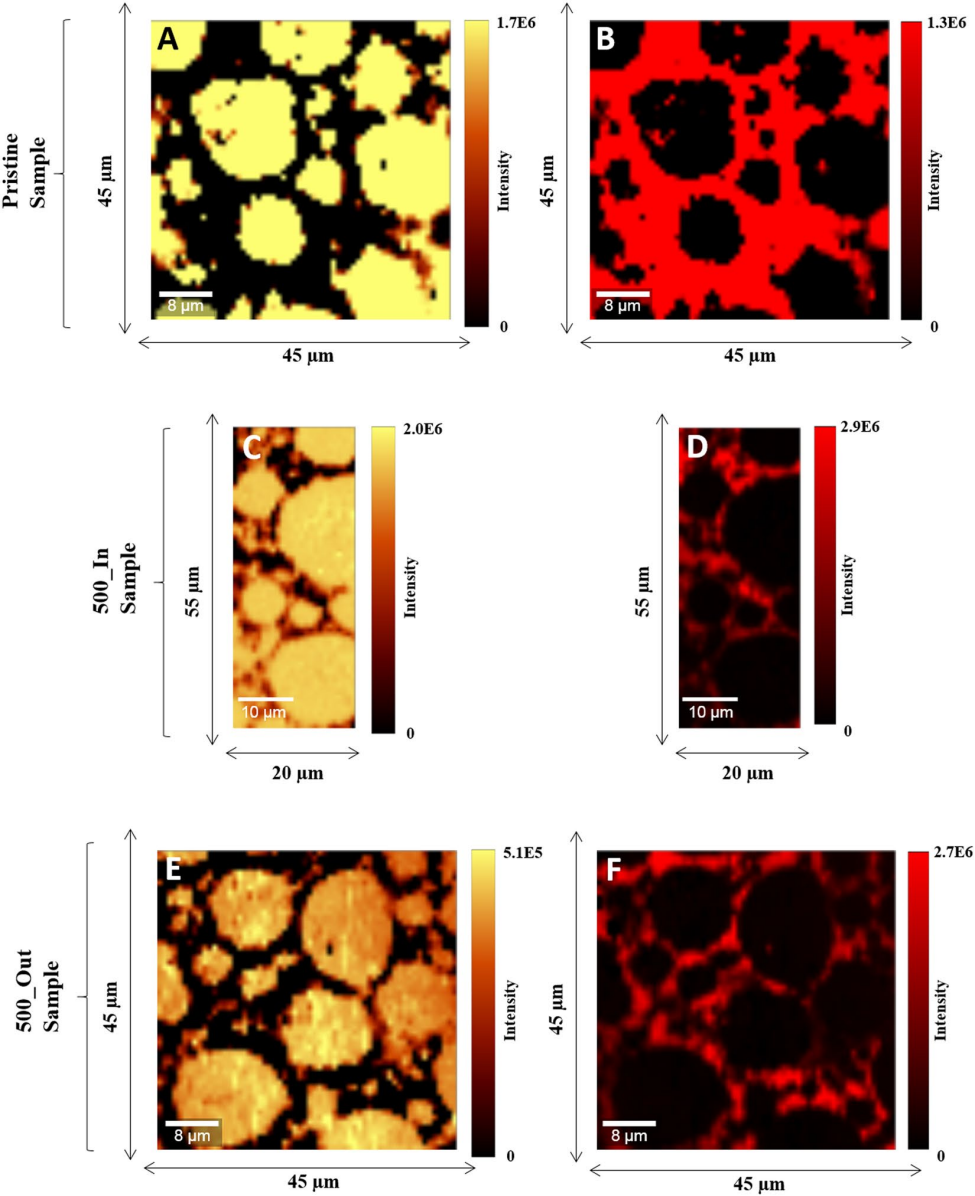
The univariate analysis of Raman spectral dataset analysis based on human intelligence. The human intelligence identifies the existence of two components, namely carbon and LiMO_2_ (M = Ni, Mn, Co), irrespective of any LIB samples.

The charge/discharge cycle dependency of capacity retention in LIB cell is shown in Fig. S5(a). The capacity retention is plotted against square-root of cycle number. The capacity retention inside the LIB cell decrease with an increase in the charging/discharging cycles and, until 300 cycles, the drop in capacity retention curve is proportional to the square-root of cycle number. Beyond 300 cycles, the capacity retention rate no longer follows the square-root law and drop much quickly, possibly, due to the side reaction inside the LIB cell. The resistance inside the cell also increases as the charging/discharging cycle progresses (Fig. S5(b). To investigate the effect of side reaction on to cathode electrode before and after the charging/discharging of the of LIB cell, three LIB samples were subjected to Raman spectral mapping; (1) Pristine (without any charging/discharging), (2) 500_IN sample (after 500 cycle of charge/discharge form interior outer region, and (3) 500_Out sample (after 500 cycle of charge/discharge from outer region, respectively. The objective of sampling from two different spatial positions after 500 cycles of charge/discharge, in the LIB cell, was to investigate spatial uniformity (interior and exterior). Figure 3 shows the univariate analysis of Raman spectral dataset analysis based on human intelligence. Results show the presence of two components, namely carbon and LiMO_2_ (M = Ni, Mn, Co), irrespective of any LIB sample. Human intelligence means the hyperspectral Raman data set (X) analysis done by an expert having three years of experience in handling LIB Raman analysis.

### Raman analysis of Pristine LIB sample

A typical hyperspectral Raman image is a 3D dataset (Fig. 1). The structure of the 3D spectral hypercube X_Pristine_ (60 × 60 × 1550) contains LiMO_2_, carbon, binder, and background information. The 3D spectral hypercube X_Pristine_ was folded into a 2D matrix (X_Pristine_: 3600, 1550) by systematically placing recorded spectrum one over the other along the wavelength axis. Figure S6(a) shows the raw Raman spectral dataset plot using the 2D X_Pristine_ matrix. The signal from the main lithium and carbon peaks in the Raman data is minimal in contrast to the cosmic noise and fluorescence contributed by the background. It is because the Raman spectrum is acquired using the CCD detectors, and the detectors often suffer from random thermal noise and spikes caused by cosmic noise. Thermal noise causes the background signature is less severe as compared to the “fluorescence” caused by the intrinsic binder of LIB cell, leaving a baseline trail. Additionally, the spikes overlay the band of interest had a larger peak area; it can potentially screw the data analysis results. The baseline correction was done before the despicking (cosmic removal). An adaptive iteratively reweighted penalized least-squares (airPLS) algorithm was used to remove the baseline from the Raman dataset^14,18^. Figure S6(b) shows the baseline corrected Raman spectral data set (X_Pristine_). It is evident from the graph that the baseline was corrected, and fluorescence was effectively removed.

For Despiking, although, there are several techniques such as wavelet processing, median/polynomial filters, and Savitzky-Golay. However, these methods often have severe constraints, given that, knowing the peak width of spikes is the prerequisite. Nevertheless, newly purposed PCA based despiking algorithm is very promising^14^. The PCA-despiking algorithm uses principal component analysis (PCA) that allows the number of variables in a multivariate data set to be reduced by retaining the crucial features and excluding the features whose contribution is either negligible or more like noise. The PCA-despiking algorithm is an ML technique that retains the essential features (i.e., scores) depending on the threshold (T) specified by the user^14^. Although the PCA-despiking algorithm works well on various kinds of dataset, nevertheless, it has a significant challenge; in particular, it fails to eliminate the spikes from the dataset in case the cosmic noise peak position for multiple spectra found to be identical. Conceding that, changing the threshold or using the algorithm in a loop does not facilitate to eliminate the cosmic noise (Fig. S7)). Predominantly, it is because the algorithm is well suited if the noise peaks are at random position, we exploited this problem for our advantage to purpose a modified version of the PCA-despiking. We have used the algorithm in the loop by alternatively transposing the dataset until all the peaks are altogether eliminated. Transposing the dataset, before calling the PCA-despiking algorithm, causes the randomness in the dataset, consequently removing all the spikes from the dataset. Figure S6(c) shows the baseline-corrected despiked Raman spectral data set (*X*_*Pristine-BD*_: 3600 × 1550). It is evident from the graph that the cosmic noise was utterly eliminated, and lithium and carbon peaks can be seen with ease.

**Figure 4:**
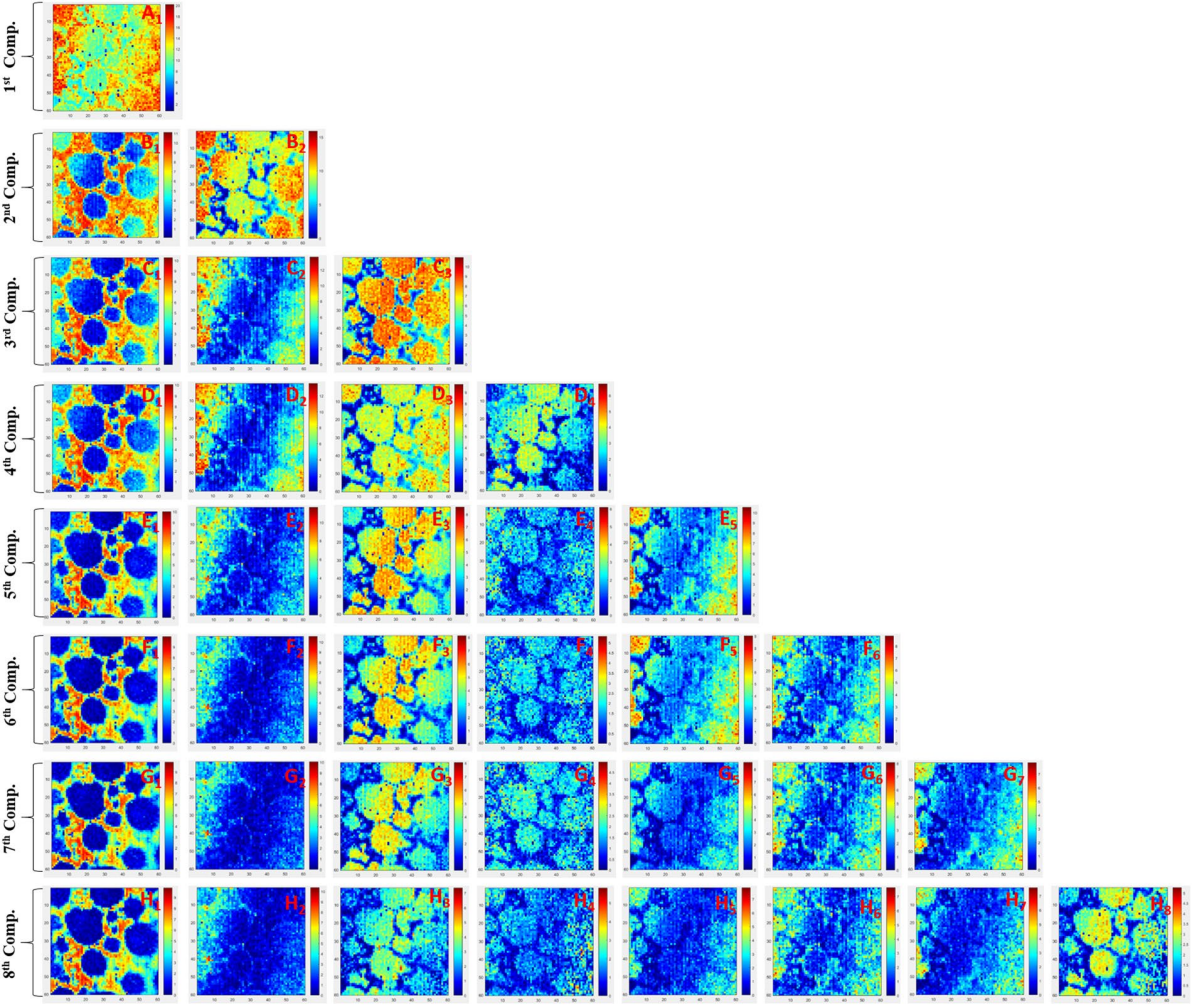
The concentration profiles (*C*_*Pristine*_, 3600 × 36) were refolded back to form an image of dimension (*C*_*Pristine*_, 60 × 60), i.e., a total of 36 concentration images. Few components repeatedly emerged, irrespective of the component selected. However, some components emerged either once or only a few times. The reliability of a component is directly proportional to its occurrence in the concentration profile data set.

Cluster-aided-MCR-ALS analysis of despiked Raman spectral data set (*X*_*Pristine-BD*_: 3600 × 1550) was done repeatedly by changing the number of components sequentially from n = 1 to *N*_*c*_ = 8. For *X*_*pristine-BD*_ LIB dataset, the total number of resulting components (*Z*) was 36 (concentration profile: *C* = 36 & spectral profile: *S*^*t*^ = 36). The concentration profiles (*C*_*Pristine*_, 3600 × 36) were refolded back to form an image of dimension (*C*_*Pristine*_, 60 × 60), i.e., a total of 36 concentration images. The refolded concentration profile and corresponding spectral profiles (*S*^*t*^_*Pristine*_, 36 × 1550) are shown in Figs. 4 and S8, respectively. As can be seen in Fig. 4, a few components repeatedly emerged, irrespective of the component selected; however, some component emerged either once or only a few times. The reliability of a component is directly proportional to its occurrence in the concentration profile data set.

Given the fact that images are more intuitive to the human brain then spectrum, the refolding of individual concentration profiles turned them into image format (CRF); sub-pixel Raman images (RCI)^1^. Motegi *et al*. have used the unfolded concentration profile for clustering^6^. However, a typical concentration profile in unfolded format (CUF) is a one-dimensional (1D) spectrum vector, and clustering such CUF is reasonably vague and provides no visual confirmation whether the rejected cluster was really worth throwing out. On the contrary, the CRF has unique advantages because the cluster can be visualized to verify the effectiveness of the clustering process and provide additional visual safeguards before discarding the cluster. The feature extraction of RCI was done using the inception algorithms, followed by the hierarchical clustering^6^. Hierarchical cluster analysis of extracted features helps to group similar images into groups called clusters.

**Figure 5:**
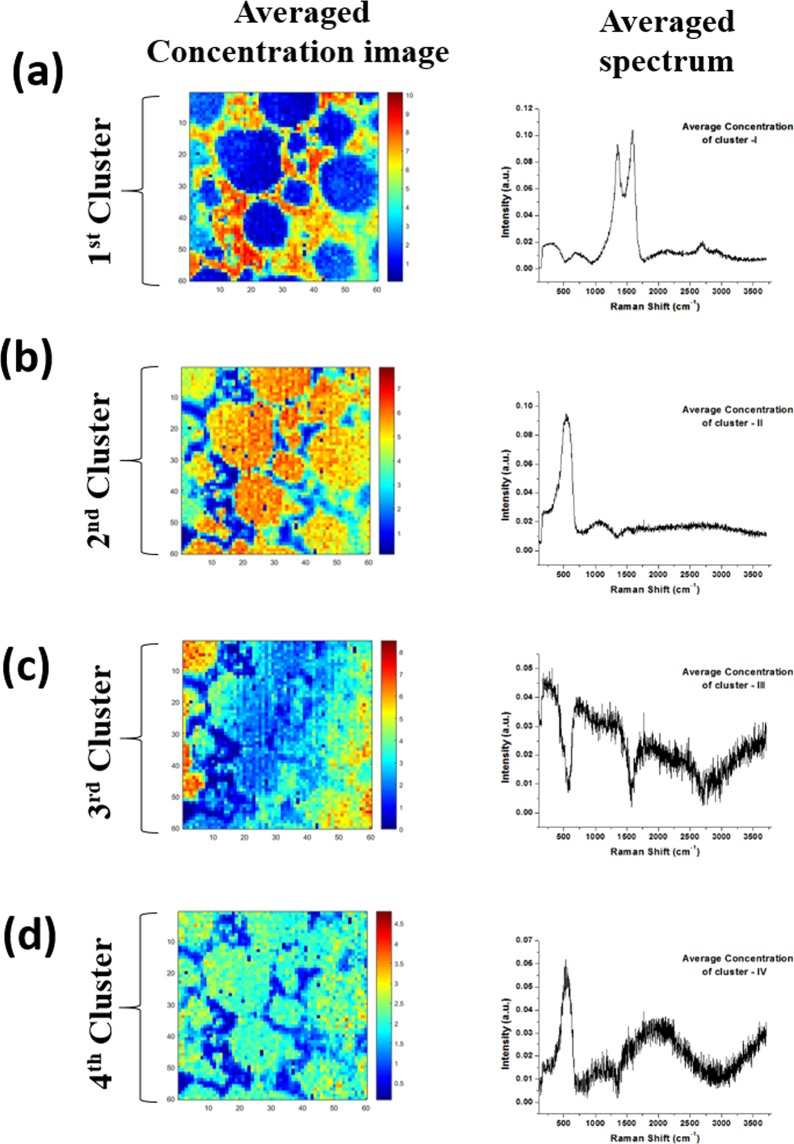
Hierarchical cluster analysis (HCA) of X_Pristine_ dataset. The averaged concentration image and corresponding spectra in the respective cluster can be seen. The clusters were assigned unique class labels depending on their spectral signature, primarily four clusters were identified; (**a**) Carbon, (**b**) LiMO_2_, (**c**) background, and (**d**) LiMO_2_ + fluorescence (LB).

Clusters with a lower correlation coefficient (<70%) and minimum leaf size <3 were rejected. Hierarchical cluster analysis was repeated with the remaining RCIs with an increase in the CC (<80%). This process was repeated until the CC reached (>90%), and beyond that, clustering does not improve. The remaining RCIs and their corresponding spectral profile *C*_*Trusted-Pristine*_ and *S*^*T*^_*Trusted-Pristine*_ are ready to be trained by NN, given the class labels are assigned to them. After the hierarchical cluster analysis (HCA), the averaged concentration image in the respective cluster is shown in Fig. 5. All the clusters were assigned unique class labels depending on their spectral signature. There are primarily four clusters; (a) Carbon, (b) LiMO_2_, (c) background and (d) LiMO_2_ + fluorescence, (Table T2 in SI). Carbon, LiMO_2_, background, and LiMO_2_ + fluorescence clusters were labeled with specific variable class name ‘C’, ‘LMO’, ‘BG’ and ‘LMO-II’, respectively and so by default, all the corresponding spectrum within a particular cluster also got the same class label. Since the dataset size of the *S*^*T*^_*Trusted-Pristine*_ is much smaller in contrast to the *C*_*Trusted-Pristine*_, *S*^*T*^_*Trusted-Pristine*_ based *NN* can be trained faster with the less computational cost. The entire analysis took around 40 minutes, starting from the background removal until the HCA (Intel-powered PC running Windows 7 with 4.0 GB of RAM).

Notably, unsupervised intelligence (C-MCR-ALS) results show that carbon and LiMO_2_ are a great match with univariate results (Fig. S9)^19^. In contrast to univariate analysis, the unsupervised intelligence extracted two additional components, namely; BG and LMO-II. The third component is the BG, where carbon and LMO microparticle can be seen evidently with a background signature that helps to ascertain the boundary between the LMO and C microparticle. The presence of the fourth component is surprising and indicates that LiMO_2_ is composed of two phases; LMO without any fluorescence and LMO-II with fluorescence.

### Training the neural network classifier (*NN*_*Pristine*_)

**Figure 6:**
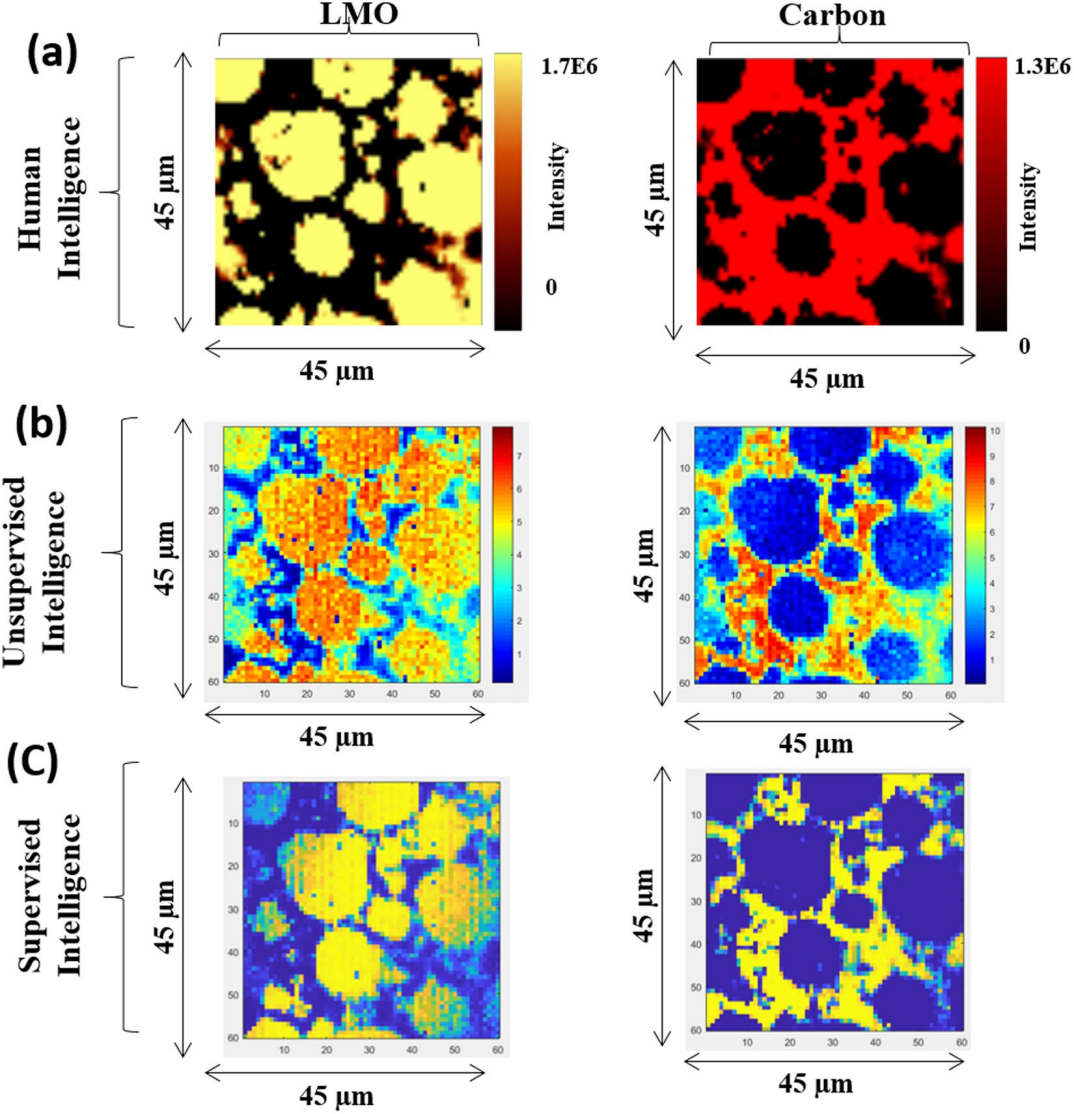
Results from three type of analytics is compared for pristine LIB sample; human, unsupervised, and supervised intelligence.

For supervised intelligence, a neural network (*NN*_*Pristine*_) classifier was trained on spectral components that were extracted by Cluster-aided-MCR-ALS analysis (*S*^*T*^_*Trusted-Pristine*_: training-set). Subsequently, the class labels of hyperspectral Raman dataset (test-set: Pristine, 500_IN, and 500_Out LIB samples) were predicted. Figure S10 shows the predicted concentration images for pristine, 500_IN, and 500_Out samples, respectively. With pristine LIB neural network model, results comparing the human, unsupervised, and supervised intelligence is shown in Fig. 6.

### Raman analysis of 500_In LIB sample

Hyperspectral Raman image for 500_In LIB sample, is a 3D spectral hypercube X_*500_In*_ (72 × 26 × 1550). The 3D spectral dataset X_*500_In*_ was folded into a 2D matrix (X_*500_In*_: 1827, 1550). Raman spectral dataset plot using the 2D X_*500_In*_ matrix is shown in Fig. S11(a). The baseline-corrected was done using Modified-PCA before the despiking (cosmic removal), baseline corrected Raman spectral data set is shown in Fig. S11(b); as a result, the baseline was corrected, and fluorescence was effectively removed. Baseline corrected despiked Raman spectral data set (X_*500_In-BD*_: 1827, 1550) shows that the cosmic noise was eliminated and LiMO_2_ and carbon peaks can be distinctly identified (Fig. S11(c)).

Cluster-aided-MCR-ALS analysis of despiked Raman spectral data set (X_*500_In-BD*_: 1827, 1550) was done repeatedly by changing the number of components sequentially from n = 1 to *N*_*c*_ = 8. For X_*500_In-BD*_ LIB dataset, the total number of resulting components (*Z*) was 36. The concentration profiles (C_*500_In*_, 1827 × 36) were refolded back to form thirty-six (36) sub-pixel RCI having a dimension (C_*500_In*_, 72 × 26). The RCI and corresponding spectral profiles (S^t^_*500_In*_, 36 × 1550) are shown in Fig. S12. Hierarchical cluster analysis (HCA) of averaged concentration image in the respective cluster is shown in Fig. S13 (we used a similar analytical pipeline, as mentioned for pristine sample analysis). Each Cluster was assigned a unique class label depending on their spectral signature. There are primarily four clusters: Carbon, LiMO_2_, LiMO_2_ + fluorescence, and background with the class label ‘C’, ‘LMO’, ‘LMO-II’ and ‘BG’, respectively (Table T3 in SI). All the corresponding spectrum within a particular cluster also assigned the same class label^15^.

The unsupervised intelligence (C-MCR-ALS) analysis depicts that carbon and LMO mapping matches exactly with univariate results (Fig. S14). Unsupervised intelligence extracted two additional components, namely ‘LMO-II and BG. Analogous to the pristine electrode, 500_IN also have LMO in two different phases (LMO & LMO-II). However, the high fluorescence coming from the core of LMO is surprising, given the fact that the binder is always attached on to the surface of the LMO microparticle^20^. However, a closer look reveals that there is little contrast within the LMO-microparticle (Fig. S15 LMO at higher magnification); it is because that some particles of the binder might have fallen on the LMO microparticle during the cross-polishing or handling of the LIB sample^2^. The fourth component is the BG, where carbon and LMO microparticle can be seen evidently with a background signature that helps to ascertain the boundary between the LMO and C microparticle. The fourth component is merely because the entire wavelength (multiple variables) was used, which is the residual imprint of data and acts as a background^1^.

### Training the neural network classifier (*NN*_*500_In*_)

**Figure 7:**
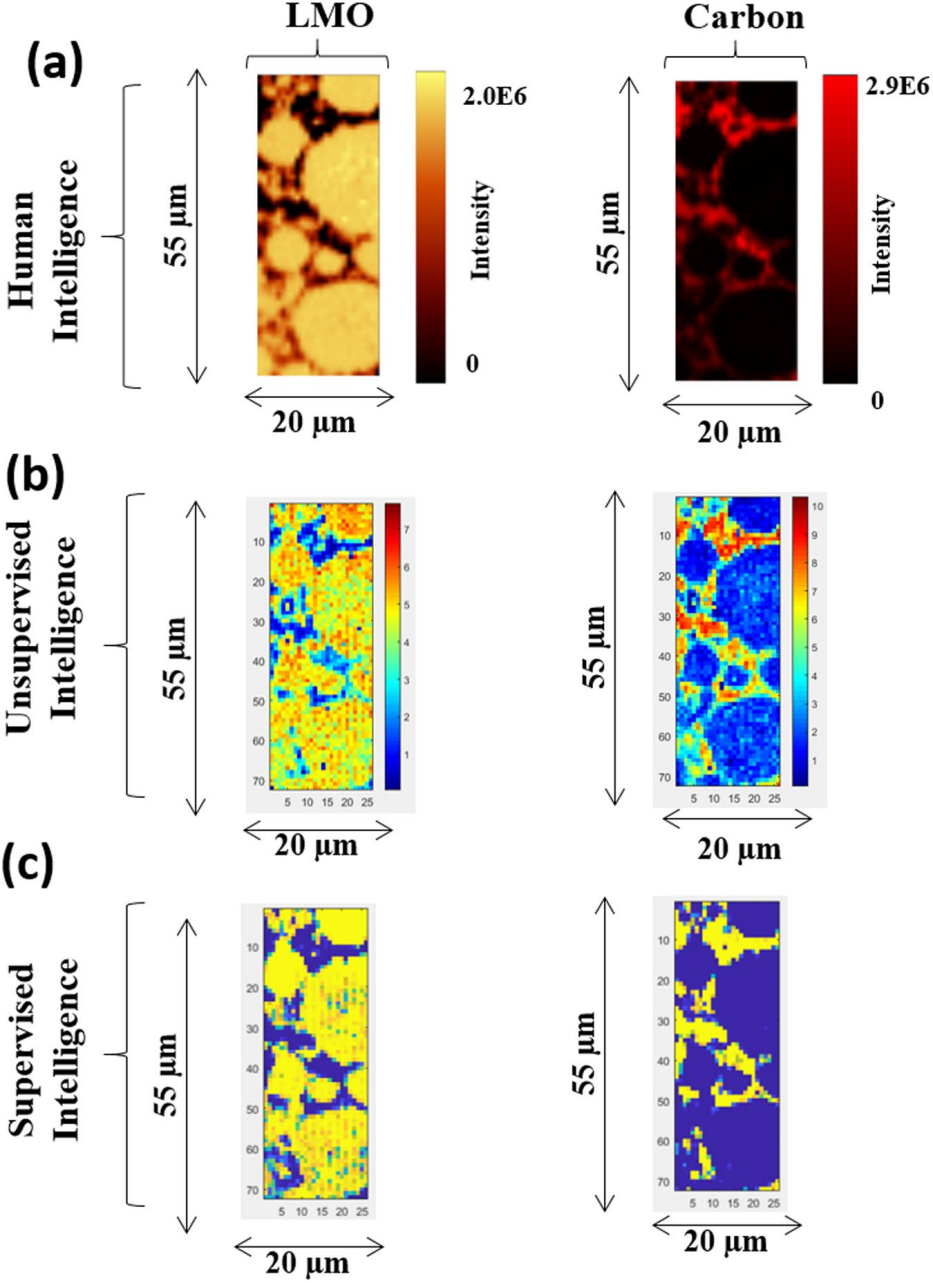
Results from three type of analytics is compared for 500_IN LIB sample; human, unsupervised, and supervised intelligence.

A neural network (NN_500_In_) classifier was trained on spectral components that were extracted by Cluster-aided-MCR-ALS analysis (*S*^*T*^_*Trusted-500_In*_: training-set). Subsequently, the class labels of hyperspectral Raman dataset (test-set: Pristine, 500_In, and 500_Out LIB samples) were predicted. Figure S16 shows the predicted concentration images for pristine, 500_IN, and 500_Out samples. With 500_IN LIB neural network model, results comparing the human, unsupervised, and supervised intelligence are shown in Fig. 7.

### Raman analysis of 500_Out LIB sample

Hyperspectral Raman image is a 3D spectral hypercube X_500_Out_ (60 × 60 × 1550). The 3D X_500_Out_ dataset was folded into a 2D matrix (X_500_Out_: 3600, 1550). Here too, a similar analytical pipeline, as mentioned for pristine sample analysis. Raw Raman spectral, baseline corrected, and baseline corrected despiked Raman spectral data set (X_500_Out-BD_: 3600, 1550) is shown in Fig. S17. It is evident from the Fig. S15 that the baseline and cosmic noise were eliminated, and LMO_2_ and carbon peaks, obviously, can be seen. Cluster-aided-MCR-ALS of a matrix (X_500_Out-BD_: 3600, 1550) was done repeatedly by changing the number of components sequentially from n = 1 to *N*_*c*_ = 8. The concentration profiles (C_500_Out_, 3600 × 36) refolded to form sub-pixel RCIs (C_500_Out_, 72 × 26 × 36) and corresponding spectral profiles (S^t^_500_Out_, 36 × 1550) is shown in Fig. S18. After HCA, averaged concentration images is shown in Fig. S19. Coincidently, four clusters were extracted; Carbon, LiMO_2_, Carbon + fluorescence (CFL), and background (Table T4 in SI) and labeled as ‘C’, ‘LMO’, ‘CFL’ and ‘BG’, respectively. All the spectrum within a particular cluster also assigned the same class label. The unsupervised intelligence (C-MCR-ALS) analysis depicts that carbon and LiMO_2_ mapping matches exactly with univariate results (Fig. S20). CFL is because of the fluorescence caused by the presence of the binder, depicting the boundary between the Li and carbon microparticle. The fourth component cannot be assigned to the LMO because the peak position is around 300 cm^−1^; the possibility is the incomplete removal of the background signature during baseline correction.

### Training the neural network classifier (NN_500_Out_)

**Figure 8:**
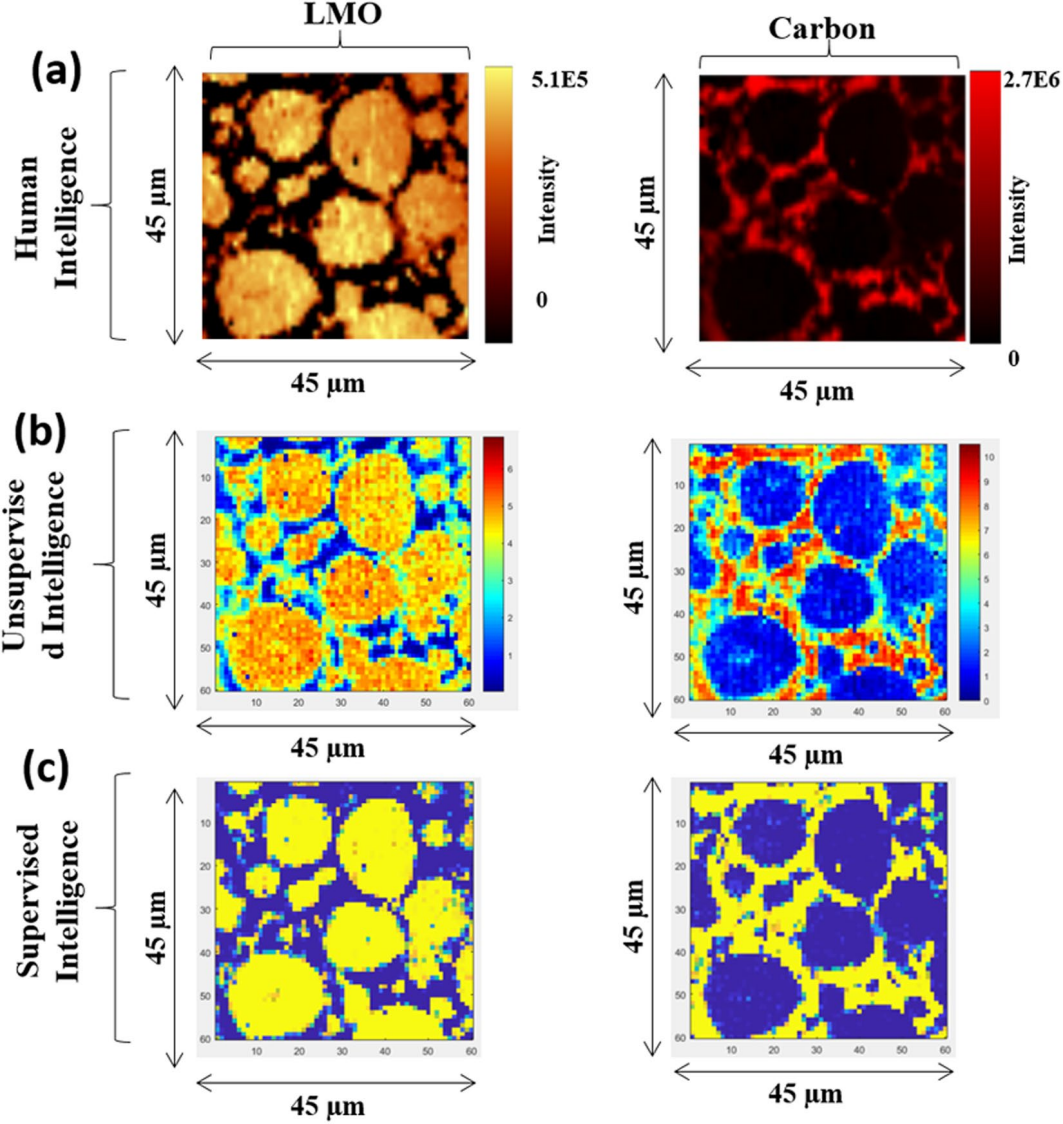
Results from three type of analytics is compared for 500_Out LIB sample; human, unsupervised, and supervised intelligence.

A neural network (NN_500_Out_) classifier was trained on spectral components that were extracted by Cluster-aided-MCR-ALS analysis (*S*^*T*^_*Trusted-500_Out*_: training-set). Subsequently, the class labels of hyperspectral Raman dataset (test-set: Pristine, 500_In, and 500_Out LIB samples) were predicted. Figure S21 shows the predicted concentration images for pristine, 500_Out, and 500_IN samples. With 500_Out LIB neural network model, results comparing the human, unsupervised, and supervised intelligence are shown in Fig. 8.

In order to understand how significant the clustering-aided MCR-ALS verses, conventional MCR-ALS analysis is, the results are illustrated in Fig. S22. Cluster-aided-MCR-ALS allowed the convoluted information content to be extracted according to the hypothesis that the original data can be reconstructed from a limited number of significant factors that are trustworthy and reproducible, irrespective of the number of components selected for extraction from the raw data^6^. The hierarchical clustering threshold removed the outliers, i.e., non-trusted spectral signature. Silhouette-clustering was performed while gradually increasing the number of components from 1 to *N*_*c*_ = 8 for pristine LIB Raman dataset. The Silhouette-clustering illustrate as the number of components changes the different number of segmentation emerges (Fig. S22(a))^18^. On the contrary, Silhouette-clustering of all four clusters (C, LMO, Background, and LMO-II extracted by Cluster-aided-MCR-ALS) results into two distinct segmentation (Fig. S22(b)); (i) trusted spectral signature, (ii) the background. The existence of the two distinct segmentation indicates that there is only a singleton spectral signature and validate the fact that the extracted components are trustworthy.

From the preceding sections, we have seen the advantage of the AF, which was designed explicitly for the LIB analysis. The analytical framework is not idiosyncratic, and it can be applied to any spectral dataset from other instrumentation as well. Cluster-assisted MCR-ALS analysis helped to resolve the genuinely reliable spectral signatures (*Z*_Trusted_) and concentration components (*C*_Trusted_) in the hyperspectral Raman datasets. Clustering and labeling the RCIs in Raman dataset has not only provided the visual confirmation for validation of the MCR-ALS results, besides it has safeguarded throwing any vital information by choosing the CC (threshold) with better objectivity. Spectral signatures (*Z*_Trusted_) were more fruitful than concentration profile (*C*_Trusted_). Firstly, the reference of extracted spectrums can be searched in the spectral database^15^. Secondly, the number of extracted spectral signatures were few in numbers, smaller in size, as a consequence, training the neural network on cluster-assisted MCR-ALS labelled data was computationally inexpensive, faster and extendable on to the enormous dataset, makes it viable for inline analysis in real-time (Quantitate analysis of LIB electrodes is discussed in SI)^3^. End-to-end AF pipeline from baseline-removal till *NN* analysis took 45 minutes. Finally, the prediction of labels was instantly within 2 seconds, and the accuracy of the network was >94%, irrespective of using any trained NN (*NN*_*Pristine*/_*NN*_*500_In*/_*NN*_*500_Out*_). It shows the effectiveness of the trained network in predicting the class labels for real-time Raman analysis with almost no human assistance.

### Capacity degradation analysis of LIB electrodes

Although, the identification of the main components (Carbon and LMO) become very intuitive using cluster-aided-MCR-ALS, irrespective of all the Raman dataset (Pristine, 500_In, and 500_Out). Nevertheless, the trace amount of components other than (C and LiMO_2_) were not consistent across all the three Raman dataset; it is because all three LIB samples contain different amount of degradation^21^. Figure S23 shows the LMO and carbon spectrum, respectively, extracted using cluster-aided-MCR-ALS analysis from pristine, 500_In, and 500_Out LIB samples. The state of charge can be qualitatively analyzed by comparing the LiMO_2_ signature^19–22^. The LIB electrode has only three transition metal ions, so there is a possibility of three *A*_*1g*_ and three *E*_*g*_ modes (Table T5 in SI). For pristine LIB sample, LMO spectra have a broad symmetric peak. The peak deconvolution shows the spectra are composed of four main peaks at 458, 524, 594, 635 cm^−1^ can be assigned to Ni (*E*_*g*_), Co (*A*_*1g*_), Mn (*E*_*g*_), and Mn (*A*_*1g*_), respectively (SI Table T6). The ration of Co (*A*_*1g*_) and Mn (*E*_*g*_) (retention coefficient) provide the state of health of battery; i.e., capacity retention. After 500 cycles, the retention coefficient reduces dramatically, and Mn (*E*_*g*_) peak gets more intense as the cycling of LIB progresses (Fig. S24). ICP results show that the stoichiometric ratio in the LIB cell was Ni: Mn: Co (5.03: 3.0: 1.97). The Raman bands are due to the motion of oxygen atoms only, M-O stretching, and O-M-O bending modes. Raman active modes of LiMO_2_ can be described as follows:

1 $$T=2{A}_{2u}+2{E}_{2u}+{A}_{1g}+{E}_{g}$$

The Raman bands for 500_IN and 500_out samples are listed in Tables T7 and T8 in SI. Given the fact, Mn is electronically inactive, and it only takes part in electronic charge transfer with Ni cations. The cation mixing between nickel cobalt and lithium ions is feasible because ionic radii of Li^+^, Ni^2+^, and Co^2+^ are very close to each other 0.76, 0.69, and 0.65A, respectively. Cation mixing is responsible for the loss of capacity and reduces lithium diffusion^19,20^. In the present study, Raman active modes were found to be broader before charge/discharge cycling and become narrower after the LIB cycling. The chances of cation mixing in the early stage of charge/discharge are higher and drastically reduces as the charge/discharge cycle progresses because of the parasite reactions. As can be seen in Fig. S24, the pristine LIB sample has a broader Raman peak compared to the 500_In and 500_Out LIB samples, notably, the Raman peak for 500_Out LIB sample has much narrow Raman peak than 500_In LIB sample, and it indicates that the interior and exterior electrode have experienced different amount of side reactions.

In order to cross-validate the cluster-assisted MCR-ALS results, pristine, 500_In, and 500_out LIB Raman datasets (baseline and despiked dataset without normalization) were also processed using NMF-SO-ARD. The results show that the LMO signature extracted by NMF-SO-ARD has close resemblance with cluster-assisted MCR-ALS signature (Fig. S25). The cluster-assisted MCR-ALS and NMF-SO-ARD results complement each other. It is recommended that the ration of Co (*A*_*1g*_) and Mn (*E*_*g*_) (retention coefficient) can provide the state of health of battery; i.e., capacity retention. Aiming the quantitative analysis of LIB electrodes, the elemental composition was evaluated for LMO and carbon. Each pixel (Fig. S26) contains the concentration of LMO and carbon. The number of pixels with an appropriate threshold is used as a metric for LMO and carbon quantification and was found to be within the range prescribed by the manufacturer of LIB electrodes.

## Conclusion

We demonstrated that the analysis of hyperspectral Raman LIB electrodes was autonomous with almost no human assistance. Modified-PCA cleansed the cosmic noise efficiently, consequently, avoid the erroneous quantitative analysis. NMF-SO-ARD algorithm was well suited for automatically identifying the maximum number of components to be fitted for the LIB hyperspectral Raman dataset. For MCR-ALS analysis, the refolding of the concentration signatures into image brought the visual confirmation and safeguarded throwing any vital information; i.e., more intuitive. Unsupervised analytics “Cluster aided MCR-ALS” has helped to extract additional components that were not identified by univariate analysis. For inline real-time analytics, the results must be instant and that it was made possible by bridging the gap between the unsupervised and supervised analytics. Cluster aided MCR-ALS analysis helped to label the reliable signatures; as a result, the trained NN helped to predict the class labels with accuracy higher than 94.0%. For the LIB electrodes, the interoperability of the NN model was found to be consistent for its major constituents, such as C and LMO. The degradation of the LIB electrode can be quantified by monitoring the retention coefficient; the ratio between Co (*A*_*1g*_) and Mn (*E*_*g*_). The present analytical framework is not idiosyncratic and could be utilized for molecular variations containing spectroscopic dataset.

## Supplementary information

Supplementary information
