# Supplementary material for: Machine Learning based Analytical Framework for Automatic Hyperspectral Raman Analysis of Lithium-ion Battery Electrodes
Source: Sci Rep. 2019 Dec 3;9:18241. doi: 10.1038/s41598-019-54770-2 (PMC6890635; doi:10.1038/s41598-019-54770-2)
Supplement: Supplementary file 1 — Supplementary information [file 41598_2019_54770_MOESM1_ESM.pdf]

Machine Learning based Analytical Framework for Automatic Hyperspectral Raman Analysis of Lithium-ion Battery Electrodes

Ankur Baliyan[1] and Hideto Imai[1]

NISSAN Analysis and Research Centre[1] (NISSAN ARC, LTD.), 1, Natsushima-cho, Yokosuka, Kanagawa, Japan, 237-0061.

Contents

1. Optical images of the LIB electrode
Figure S1.

2. Illustration of modified PCA-despiking algorithm.
Figure S2.

3. Flow chart illustration of cluster-aided C-MCR-ALS process to identify the reliable clusters.
Figure S3.

4. Illustration of the NN architecture.
Figure S4.

5. Charge/Discharge of LIB cells.
Figure S5.

6. Despiking and removal of cosmic noise from the hyperspectral Raman Dataset ($X_{Pristine}$).
Figure S6.

7. Conventional airPLS algorithm tested on random dataset.
Figure S7.

8. Concentration profiles extracted by cluster-aided-MCR-ALS analysis of $X_{Pristine}$ dataset.
Figure S8.

9. Univariate vs Unsupervised intelligence (C-MCR-ALS) results of $X_{Pristine}$ dataset.
Figure S9.

10. In-line class labels prediction with neural network ($NN_{Pristine}$) classifier.
Figure S10.

11. Despiking and removal of cosmic noise from the hyperspectral Raman Dataset (X_{500_In}).
Figure S11.

12. Cluster-aided-MCR-ALS analysis resulted in the RCI (C_{500_In}, *1827* x *36*) and corresponding spectral profiles ($S^t_{500\_In}$, *36* x *1550*).
Figure S12.

13. Hierarchical cluster analysis (HCA) of X$_{500_In}$ dataset.
Figure S13.

14. Univariate vs Unsupervised intelligence (C-MCR-ALS) results of X$_{500_In}$ dataset.
Figure S14.

15. LMO mapping of X$_{500_In}$ at higher magnification.
Figure S15.

16. In-line class labels prediction with neural network (NN_{500_In}) classifier.
Figure S16.

17. Despiking and removal of cosmic noise from the hyperspectral Raman Dataset (X$_{500_out}$).
Figure S17.

18. Cluster-aided-MCR-ALS analysis resulted in concentration profiles (C_{500_out}, *3600* x *36*) and corresponding spectral profiles ($S^t_{500\_out}$, *36* x *1550*).
Figure S18.

19. Hierarchical cluster analysis (HCA) of X$_{500_Out}$ dataset.
Figure S19.

20. Univariate vs Unsupervised intelligence (C-MCR-ALS) results of X$_{500_out}$ dataset.
Figure S20.

21. In-line class labels prediction with neural network (NN_{500_out}) classifier.
Figure S21.

22. Silhouette-clustering on MCR-ALS extracted components and HCA clusters (pristine LIB Raman dataset).
Figure S22.

23. LMO and carbon spectrum extracted using cluster-aided-MCR-ALS analysis from pristine, 500_In, and 500_Out LIB samples.
Figure S23.

24. LMO peak deconvolution for pristine, 500_In, and 500_Out LIB samples.
Figure S24.

25. LMO and carbon spectrum extracted using NMF-SO-ARD from pristine, 500_In, and 500_Out LIB samples.
Figure S25.

26. Quantitative analysis of LIB electrodes.
Figure S26.

27. Baseline correction model.

28. (i) Table T1 - Spectra acquisition information
(ii) Table T2 - Class label information for pristine LIB electrode
(iii) Table T3 - Class label information for 500_IN LIB electrode

(iv) Table T4 - Class label information for 500_Out LIB electrode
(v) Table T5 - Deconvolution of LMO extracted from the pristine LIB sample
(vi) Table T6 - Deconvolution of LMO extracted from the 500_IN LIB sample
(vii) Table T7 - Deconvolution of LMO extracted from the 500_Out LIB sample

1. Optical images of the LIB electrode

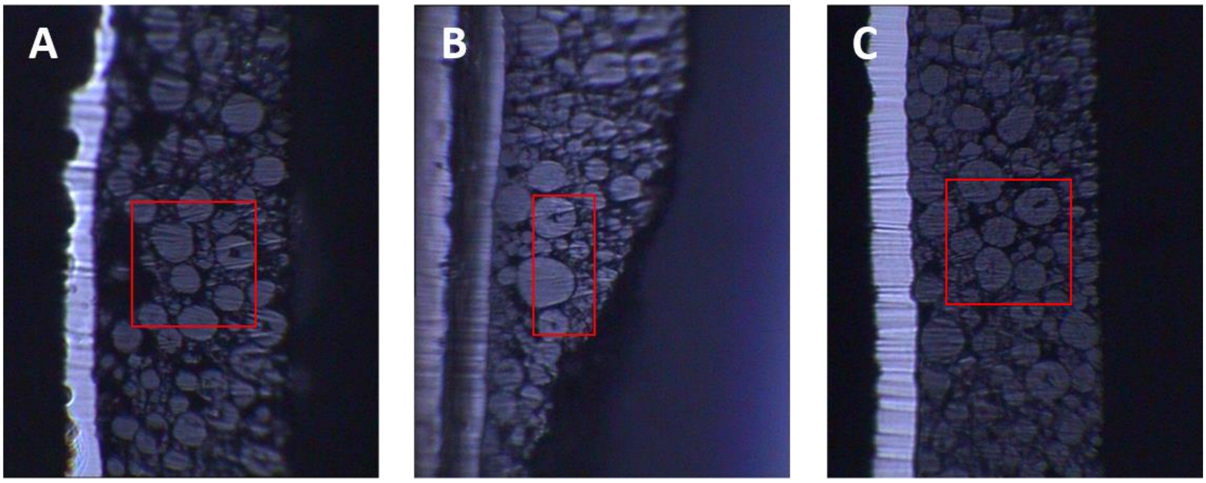

Figure S1: Optical images of the LIB electrode. Red colour box depicts the region that was used to acquire the Raman spectra. In total three LIB electrodes; **(a)** Pristine sample – without any charge/discharge, **(b)** after 500 cycles of charge/discharge – from interior region of the cathode, and **(c)** after 500 cycles of charge/discharge – from outer region of the cathode.

2. Illustration of modified PCA-despiking algorithm.

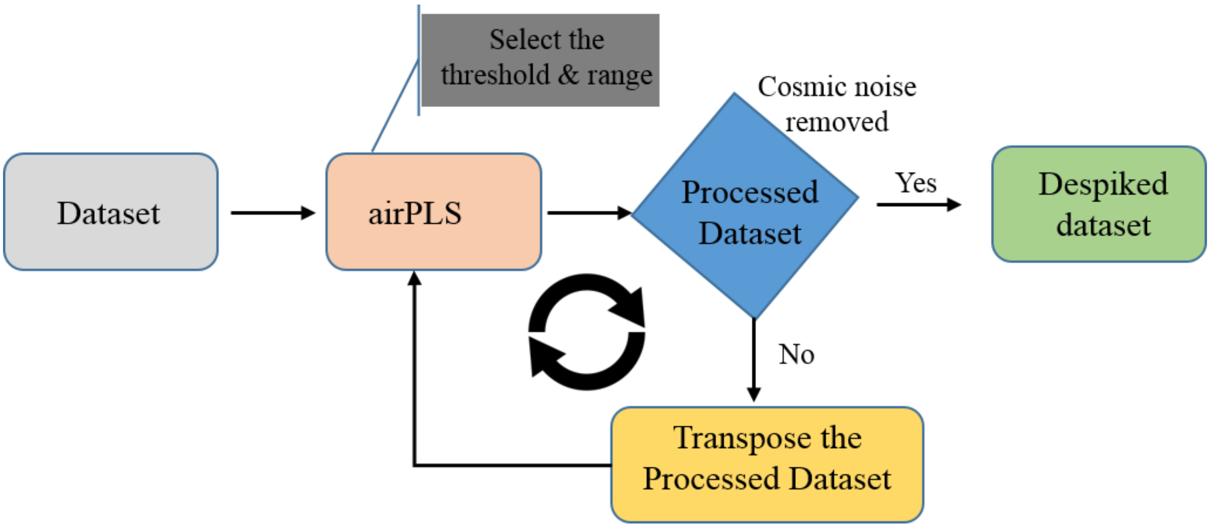

Figure S2: Illustration of modified PCA-despiking algorithm. The data-set was transpose after each iteration of PCA-despiking step.

3. Flow chart illustration of cluster-aided C-MCR-ALS process to identify the reliable clusters.

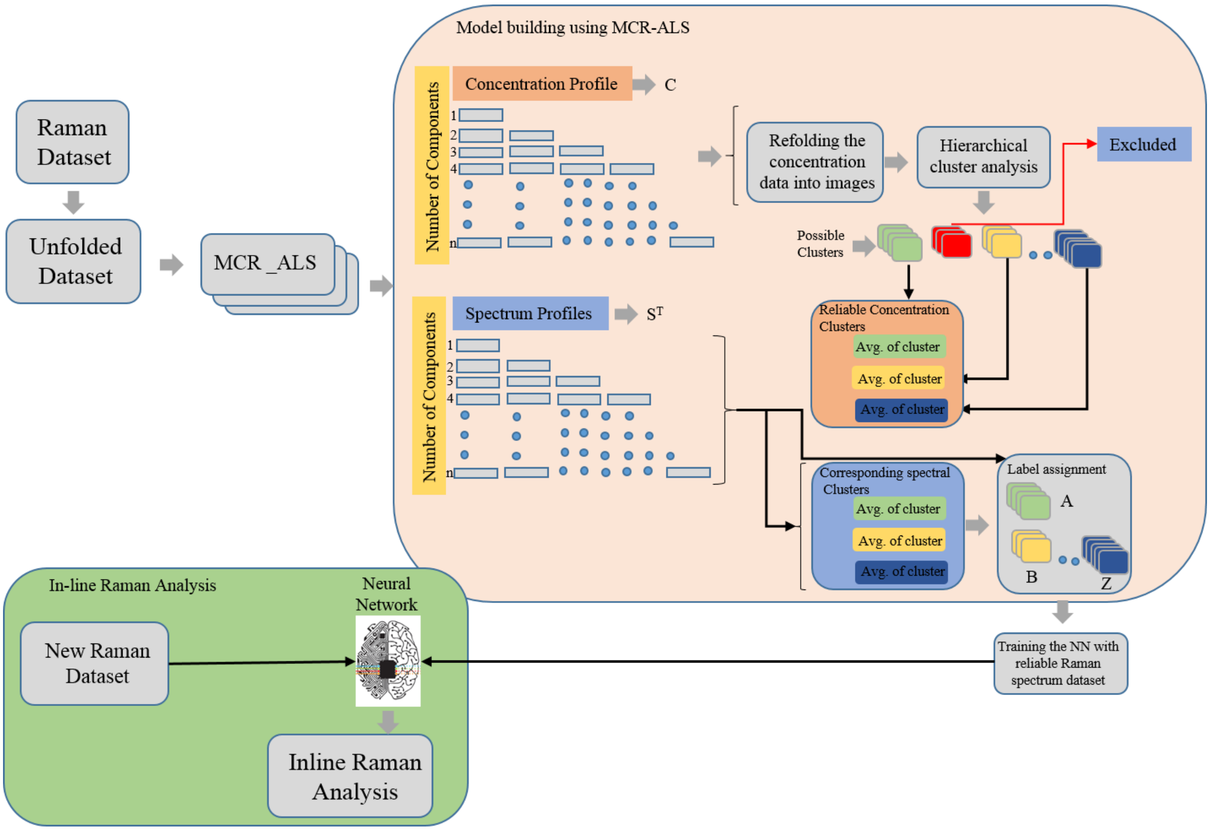

Figure S3: A flow chart illustration of cluster-aided C-MCR-ALS process to identify the reliable clusters. Here, C-MCR-ALS analysis was performed for all three LIB samples, sequentially changing n from one to $N_c = 8$. For individual LIB sample, the total number of resulting components (Z) was 36 (concentration profile: $C = 36$ & spectral profile: $S^t = 36$).

4. Illustration of the NN architecture.

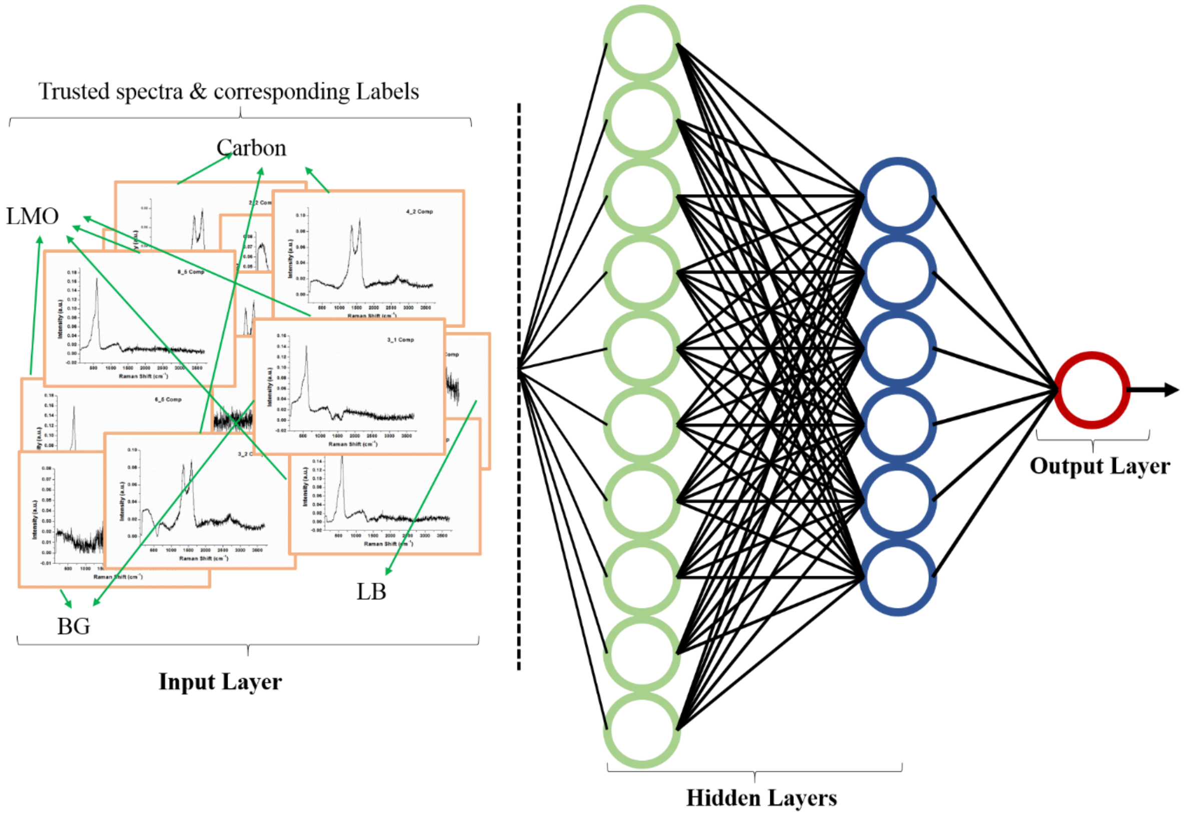

Figure S4: Illustration of the NN architecture. NN architecture is consist of four layers; one input layer, two hidden layers, and one output layer, respectively. First and second hidden layers have 10 and six perceptrons, respectively. The last layer received input from the last hidden layer of the network, and has the output variables equal to the cumulative reliable clusters identified by C-MCR-ALS analysis.

5. Charge/Discharge of LIB cells.

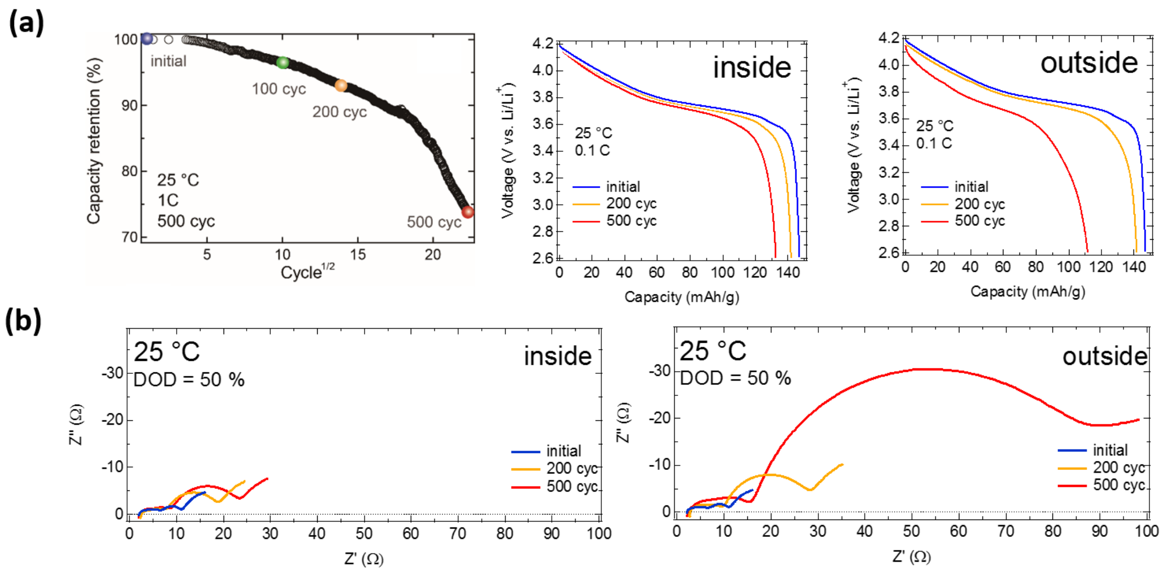

Figure S5: **(a)** The charge/discharge cycle dependency of capacity retention in LIB cells. **(b)** Electrochemical impedance spectra of LIB samples.

6. Despiking and removal of cosmic noise from the hyperspectral Raman Dataset ($X_{Pristine}$).

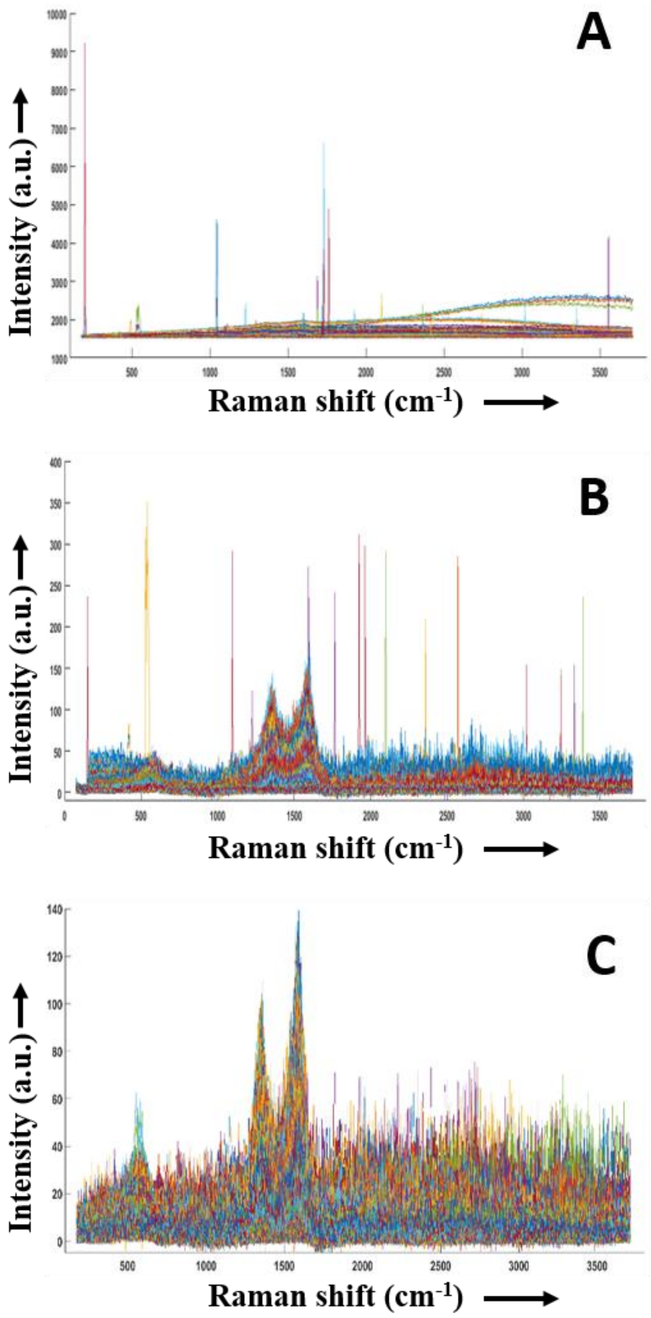

Figure S6: Despiking and removal of cosmic noise from the hyperspectral Raman Dataset ($X_{Pristine}$). **(a)** Raw Raman spectral dataset plot using the 2D $X_{Pristine}$ matrix, the signal from the main lithium and carbon peaks in the Raman data is minimal in contrast to the cosmic noise and fluorescence contributed by the background. **(b)** The baseline corrected Raman spectral data set ($X_{Pristine}$), the baseline was corrected and fluorescence was effectively removed. **(c)** The baseline corrected despiked Raman spectral data set ($X_{Pristine\text{-}BD}$: 3600 x 1550), the cosmic noise was utterly eliminated and lithium and carbon peaks can be seen with ease.

7. Conventional airPLS algorithm tested on random dataset.

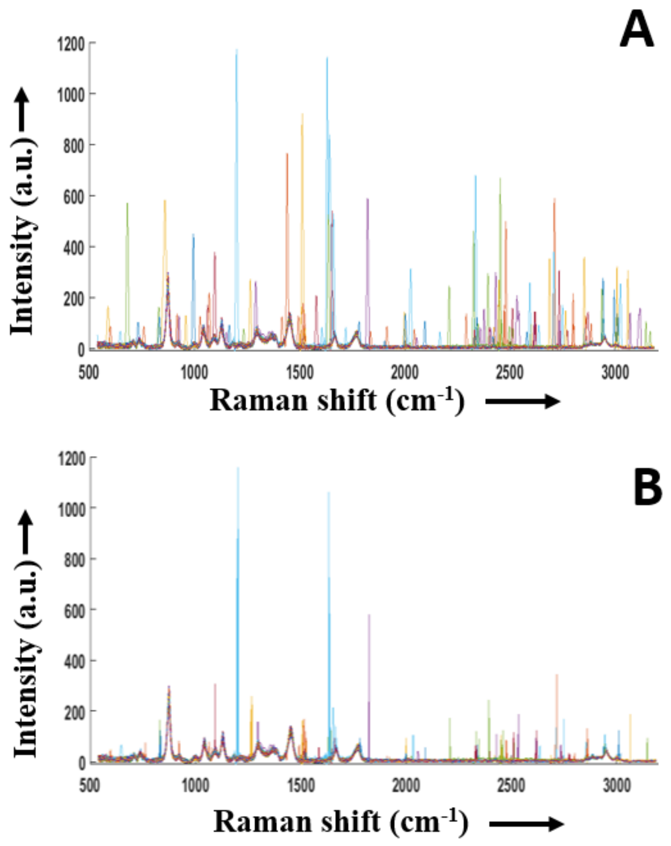

Figure S7: Conventional airPLS algorithm, changing the threshold or using the algorithm in the loop does not facilitate to eliminate the cosmic noise.

8. Concentration profiles extracted by cluster-aided-MCR-ALS analysis of $X_{Pristine}$ dataset.

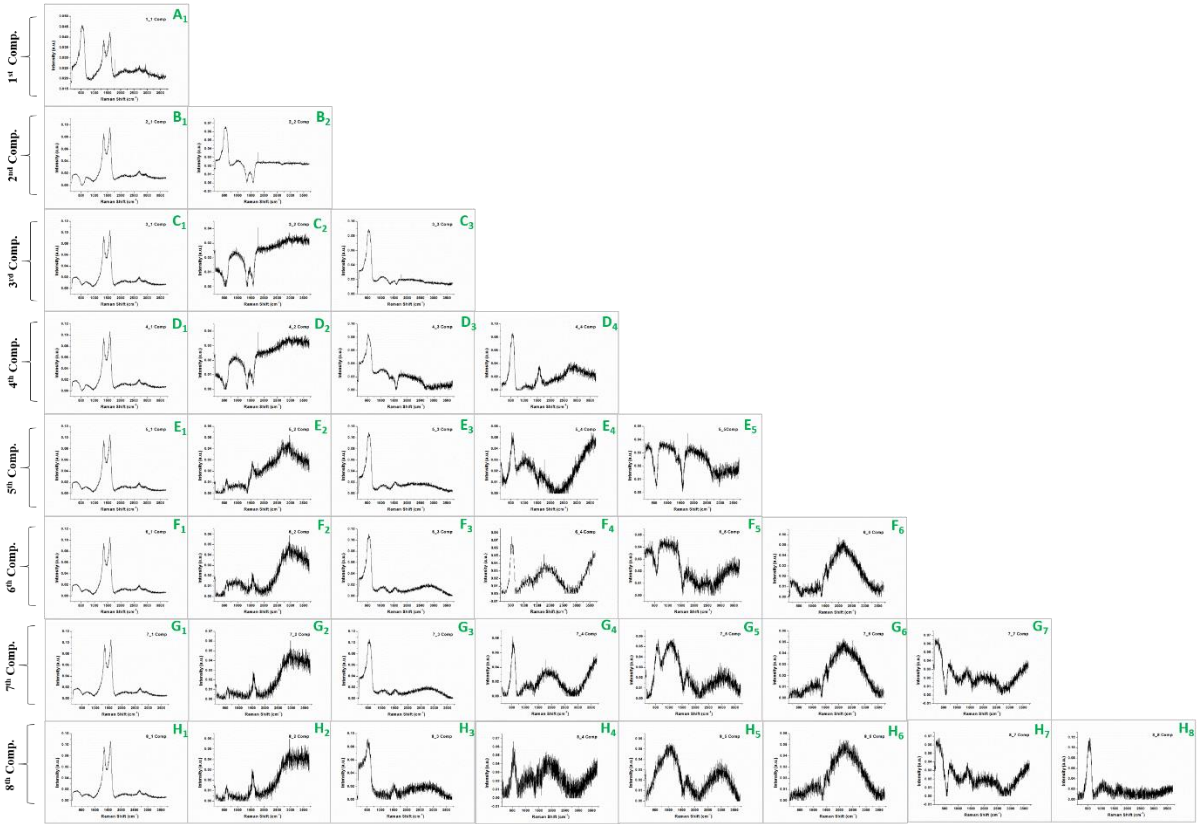

Figure S8: Cluster-aided-MCR-ALS analysis resulted in concentration profiles ($C_{Pristine}$, *3600* x *36*) and corresponding spectral profiles ($S^t_{Pristine}$, *36* x *1550*). Here, all spectral profiles ($S^t_{Pristine}$, *36* x *1550*) can be seen, however for corresponding concentration profiles, see Figure 4.

9. Univariate vs unsupervised intelligence (C-MCR-ALS) results of $X_{Pristine}$ dataset.

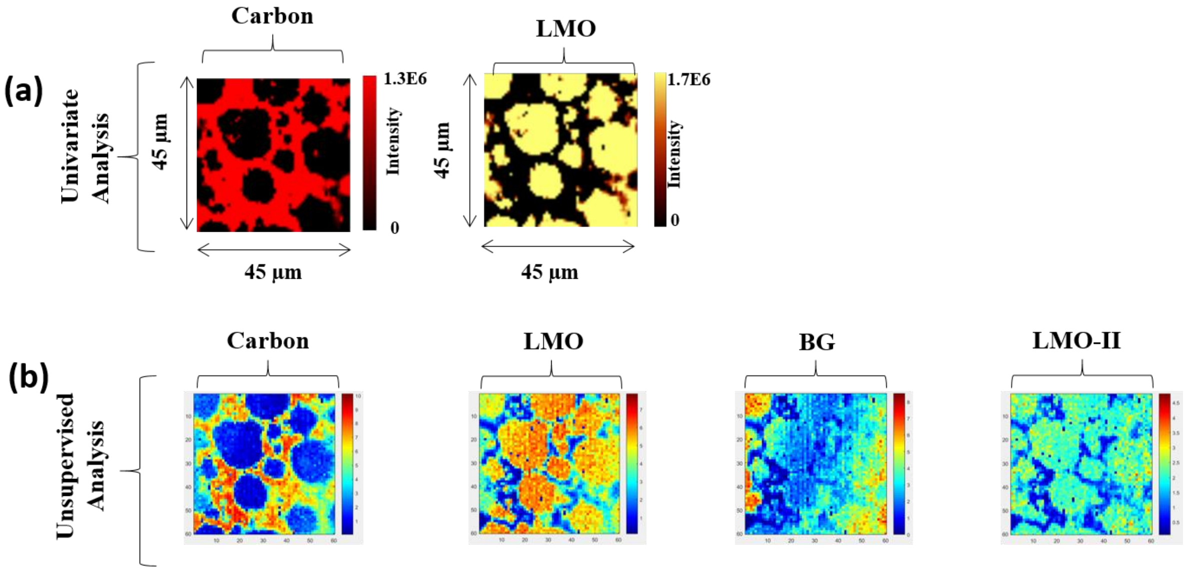

Figure S9: Univariate vs unsupervised intelligence (C-MCR-ALS) results of $X_{Pristine}$ dataset. Carbon and $LiMO_2$ are a great match with univariate results. However, supervised intelligence extracted two additional components.

10. In-line class labels prediction with neural network (*$NN_{Pristine}$*) classifier.

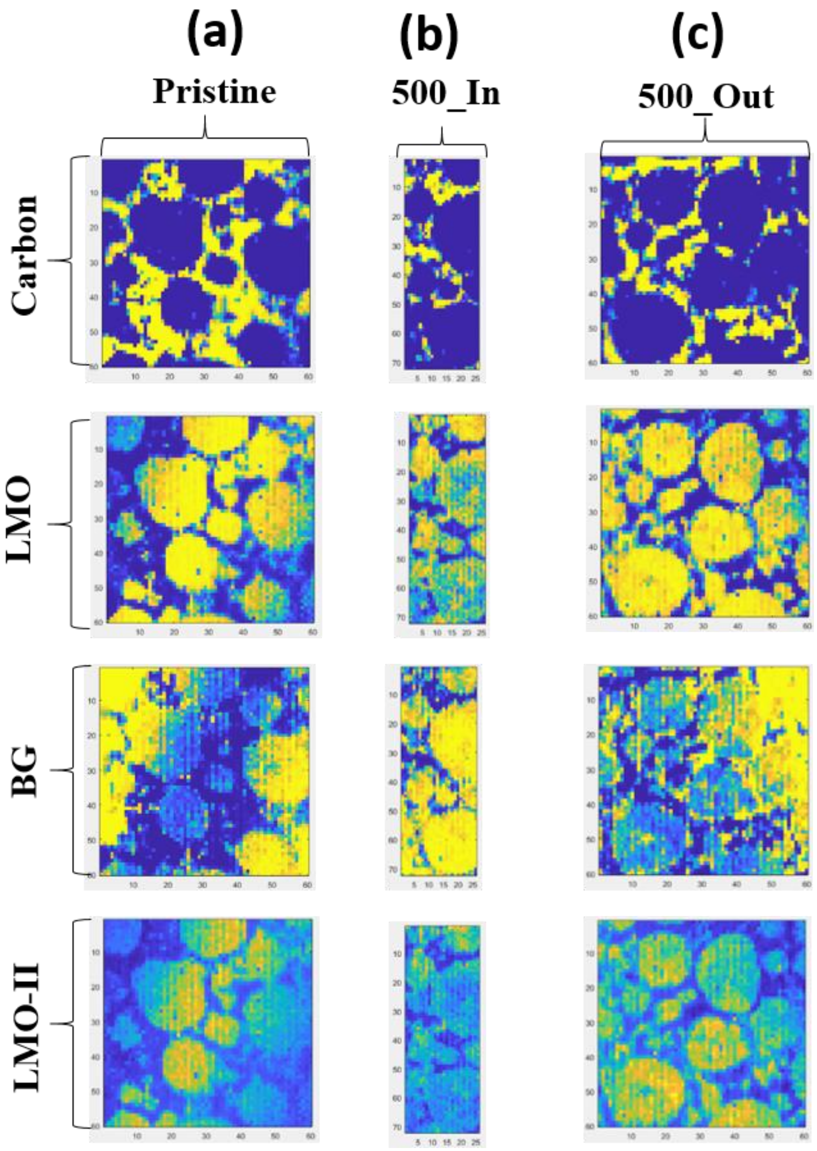

Figure S10: In-line class labels prediction with neural network ($NN_{Pristine}$) classifier. Results shows the predicted concentration images for pristine, 500_Out, and 500_IN samples.

11. Despiking and removal of cosmic noise from the hyperspectral Raman Dataset (X_{500_In}).

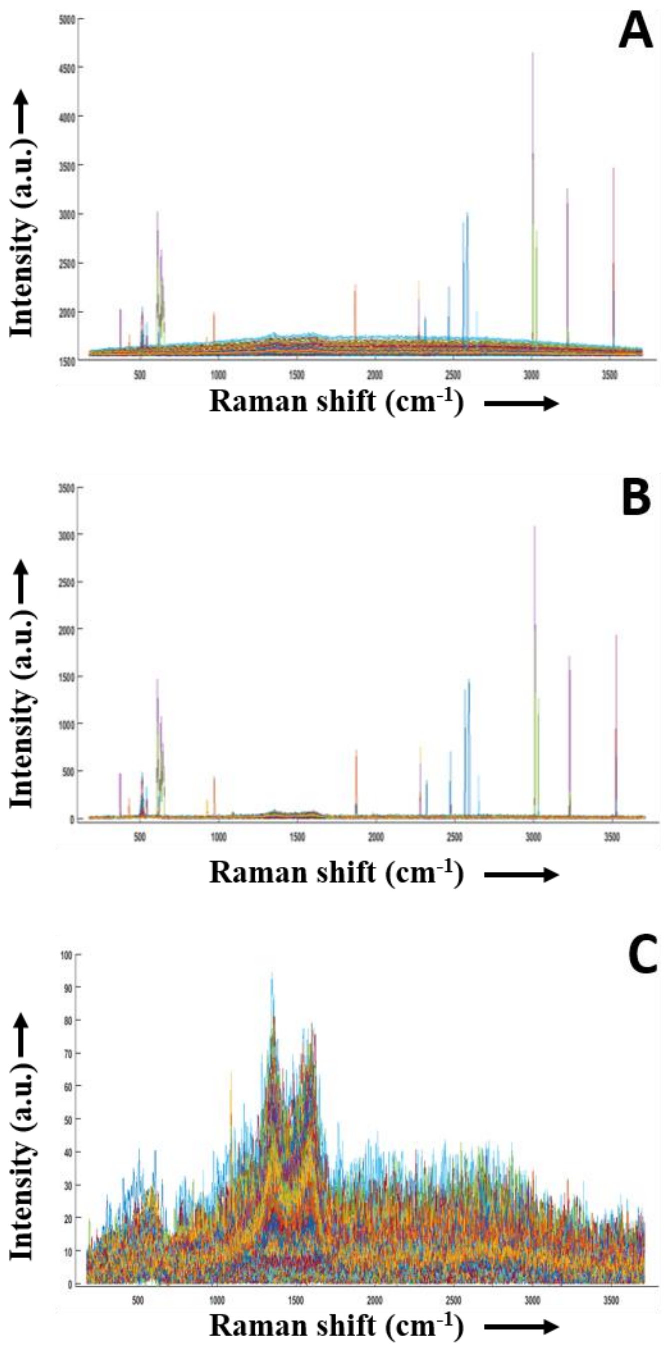

Figure S11: Despiking and removal of cosmic noise from the hyperspectral Raman Dataset (X_{500_In}). (a) Raw Raman spectral dataset plot using the 2D X_{500_In} matrix, the signal from the main lithium and carbon peaks in the Raman data is minimal in contrast to the cosmic noise and fluorescence contributed by the background. (b) The baseline corrected Raman spectral data set (X_{500_In}), the baseline was corrected and fluorescence was effectively removed. (c) The baseline corrected despiked Raman spectral data set ($X_{500\_In\text{-}BD}$: 1827, 1550), the cosmic noise was utterly eliminated and lithium and carbon peaks can be seen with ease.

12. Cluster-aided-MCR-ALS analysis resulted in the RCI (C_{500_In}, *1827* x *36*) and corresponding spectral profiles ($S^t_{500\_In}$, *36* x *1550*).

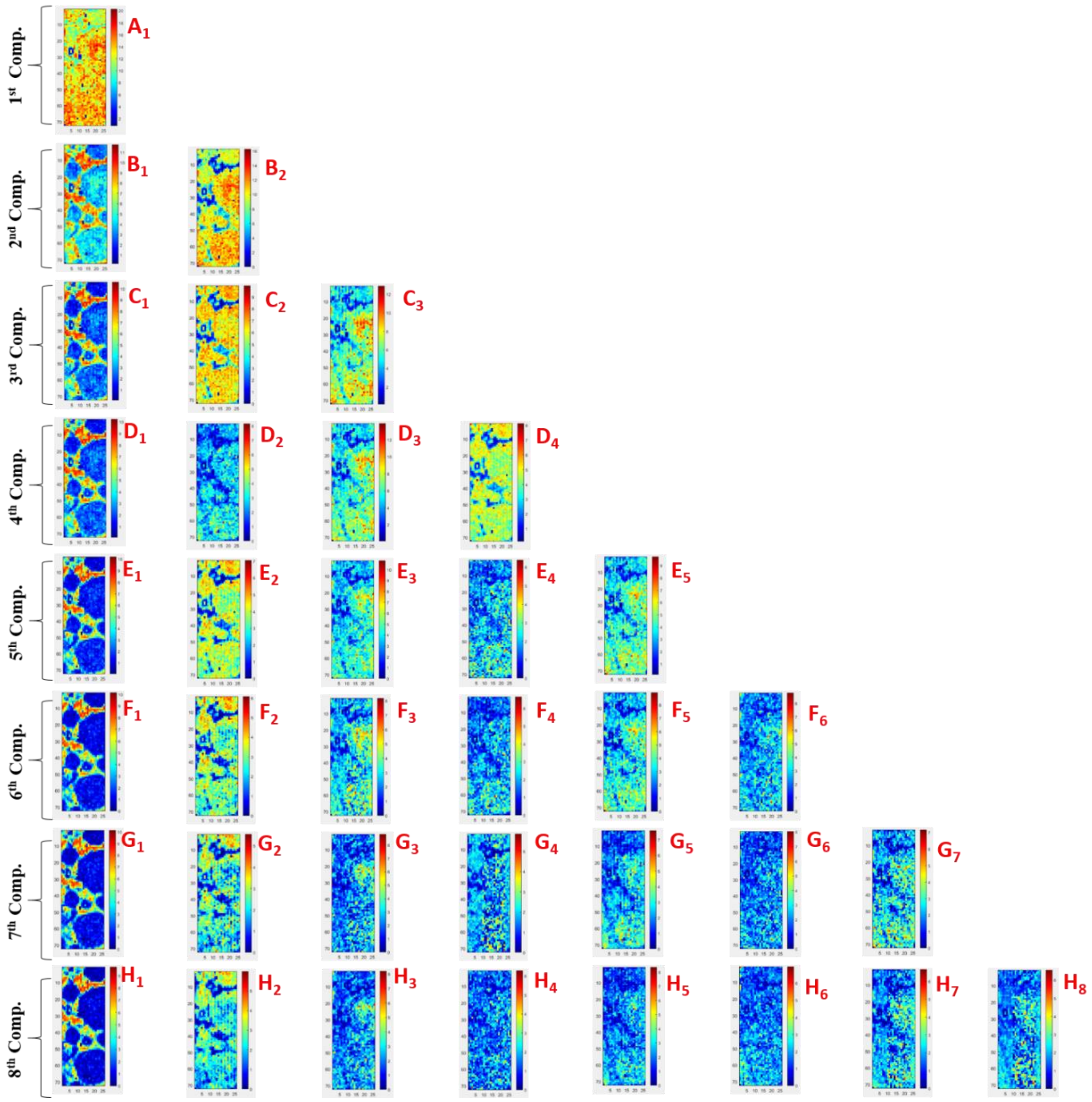

Figure S12a: Cluster-aided-MCR-ALS analysis resulted in the RCI (C_{500_In}, *1827* x *36*) and corresponding spectral profiles ($S^t_{500\_In}$, *36* x *1550*). (a) The concentration profiles (C_{500_In}, *1827* x *36*) were refolded back to form thirty-six (36) sub-pixel RCI having a dimension (C_{500_In}, *72* x *26*). (b) spectral profiles ($S^t_{500\_In}$, *36* x *1550*).

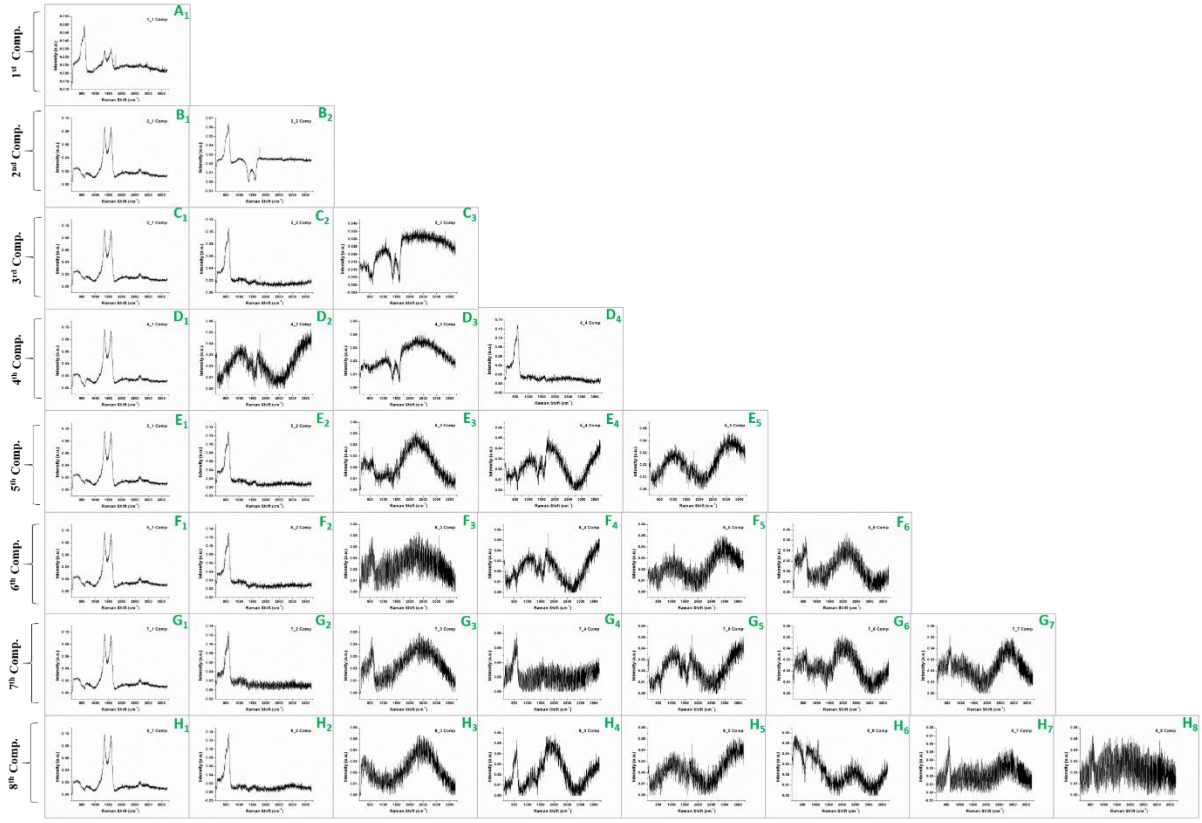

Figure S12b: Cluster-aided-MCR-ALS analysis resulted in the RCI (C_{500_In}, *1827* x *36*) and corresponding spectral profiles ($S^{t}_{500\_In}$, *36* x *1550*). (b) spectral profiles ($S^{t}_{500\_In}$, *36* x *1550*).

13. Hierarchical cluster analysis (HCA) of X_{500_In} dataset.

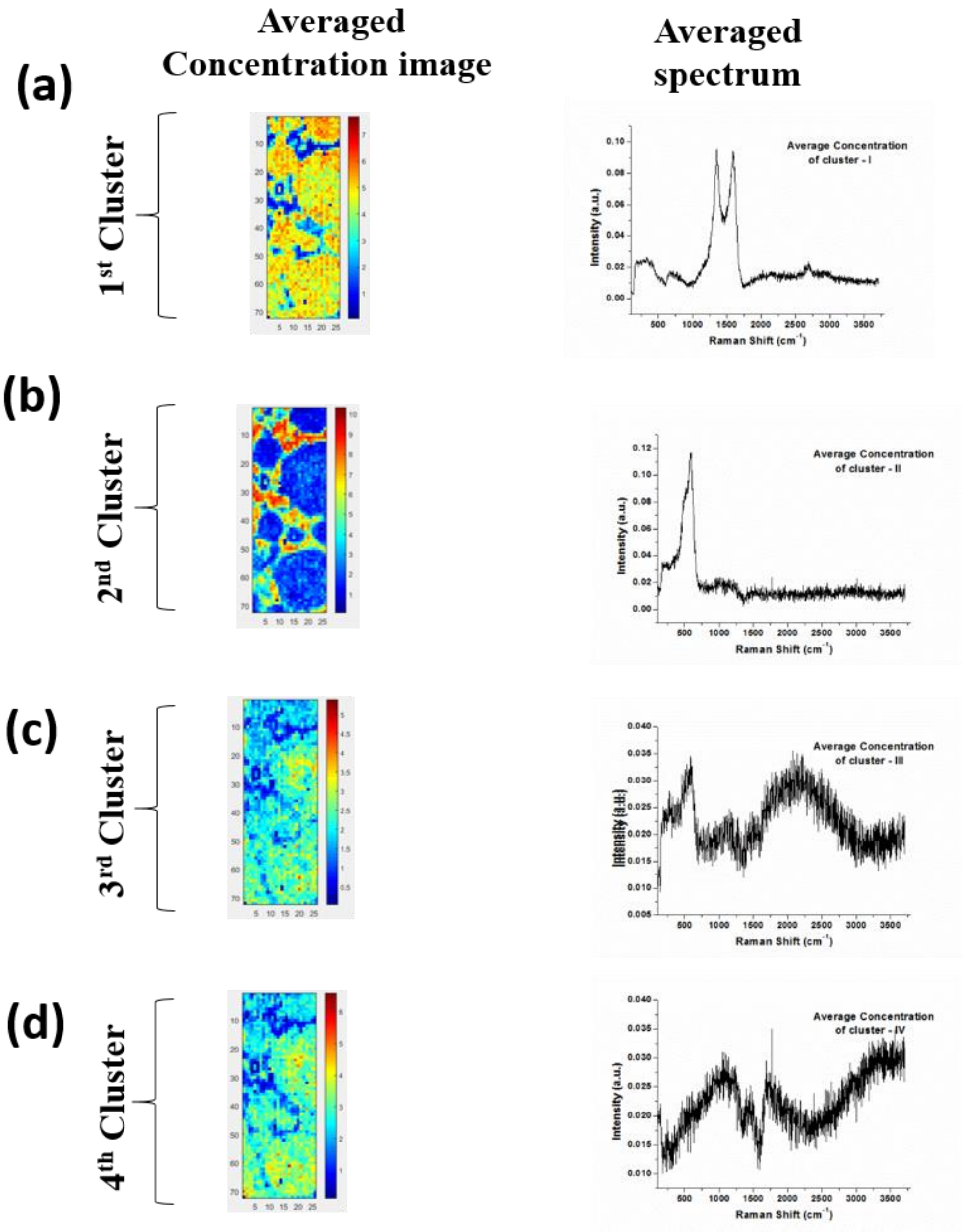

Figure S13: Hierarchical cluster analysis (HCA) of X_{500_In} dataset. The averaged concentration image and corresponding spectra in the respective cluster can be seen.

14. Univariate vs Unsupervised intelligence (C-MCR-ALS) results of X_{500_In} dataset.

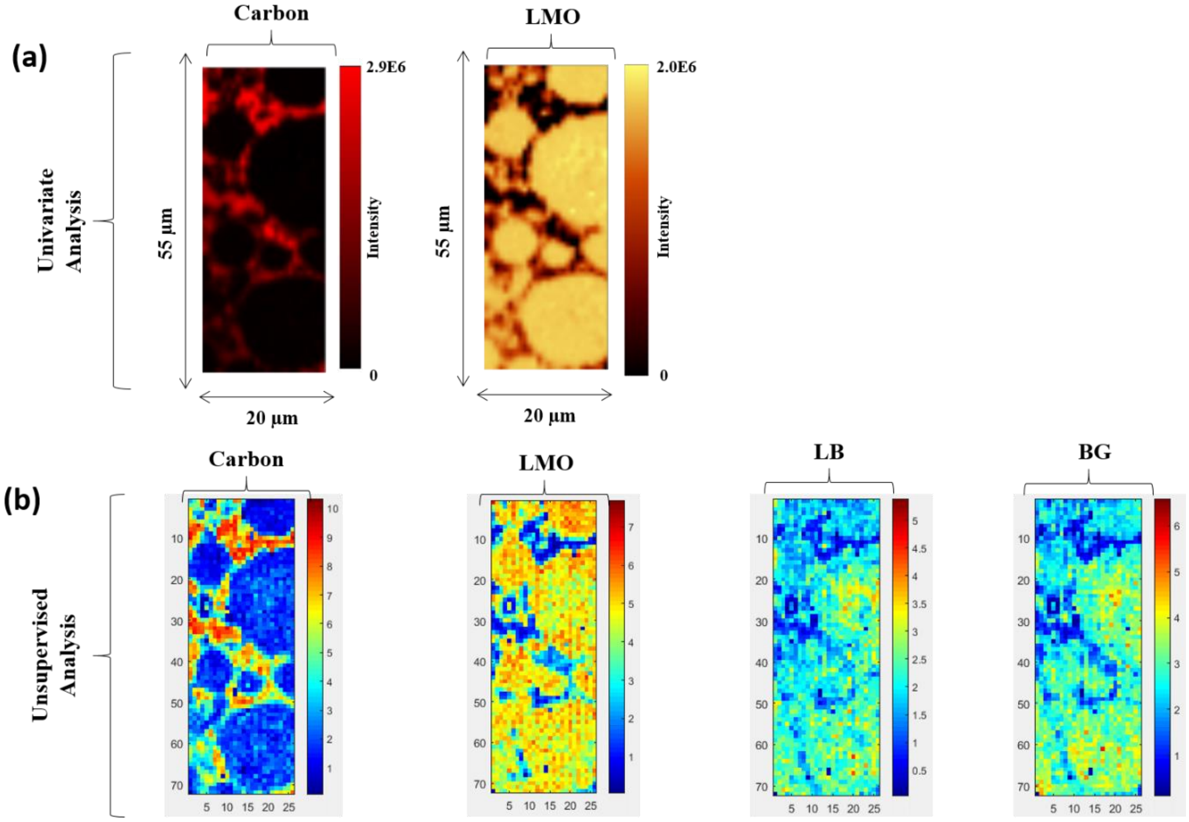

Figure S14: Univariate vs unsupervised intelligence (C-MCR-ALS) results of X_{500_In} dataset. The supervised intelligence (C-MCR-ALS) analysis depicts that carbon and LMO mapping matches exactly with univariate results.

15. LMO mapping of X_{500_In} at higher magnification.

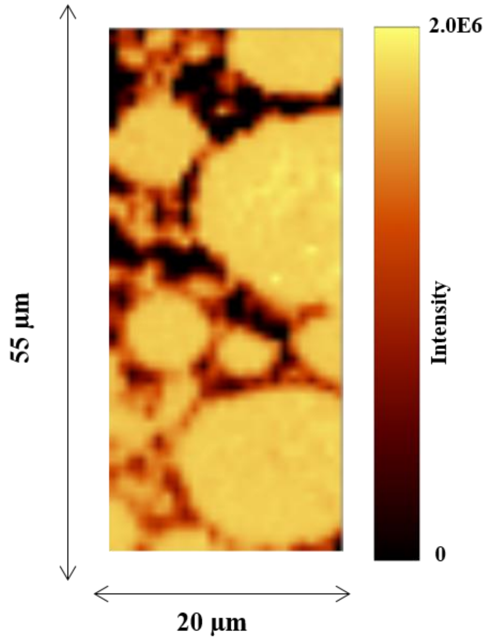

Figure S15: LMO mapping of X_{500_In} at higher magnification. The binder particle is marked with circle (redcolor).

16. In-line class labels prediction with neural network (NN_{500_In}) classifier.

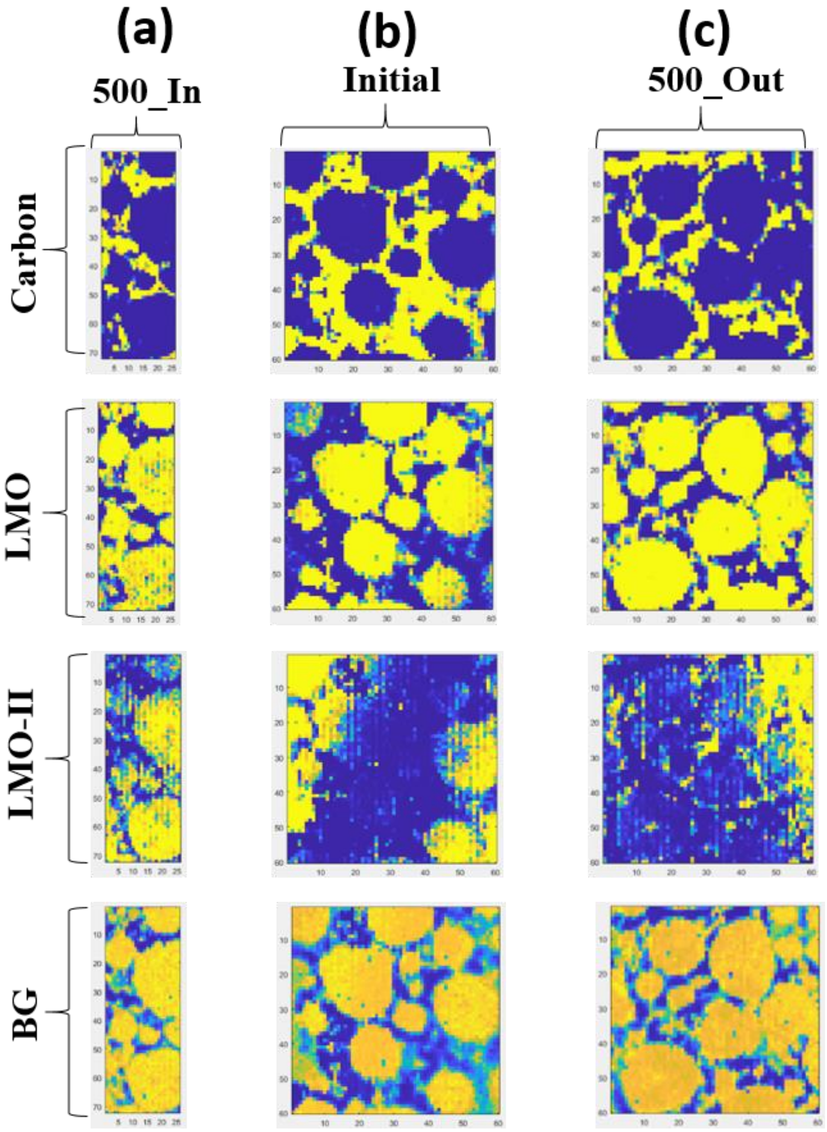

Figure S16: In-line class labels prediction with neural network (NN_{500_In}) classifier. Results shows the predicted concentration images for pristine, 500_Out, and 500_IN samples.

17. Despiking and removal of cosmic noise from the hyperspectral Raman Dataset (X_{500_out}).

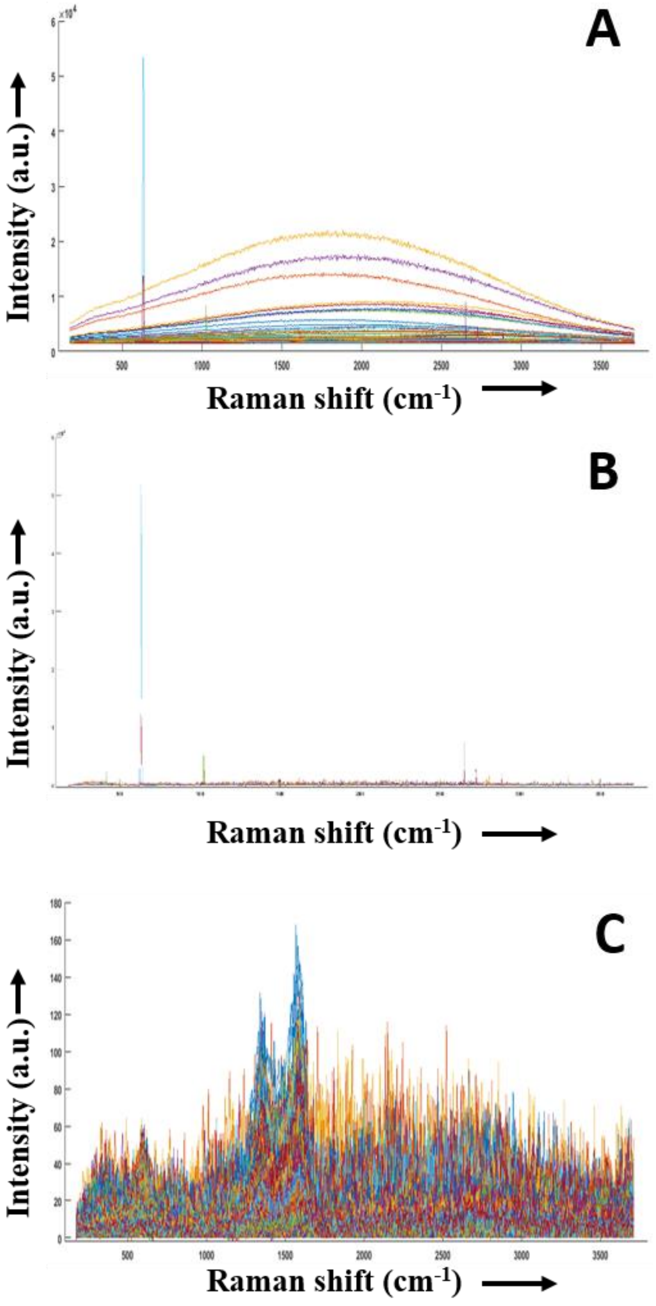

Figure S17: Despiking and removal of cosmic noise from the hyperspectral Raman Dataset (X_{500_out}). (a) Raw Raman spectral dataset plot using the 2D X_{500_out} matrix. (b) The baseline corrected Raman spectral data set (X_{500_out}). (c) The baseline corrected despiked Raman spectral data set ($X_{500\_In\text{-}BD}$: 3600, 1550), the cosmic noise was utterly eliminated and lithium and carbon peaks can be seen with ease.

18. Cluster-aided-MCR-ALS analysis resulted in concentration profiles (*C_{500_out}*, *3600* x *36*) and corresponding spectral profiles (*$S^t_{500\_out}$*, *36* x *1550*).

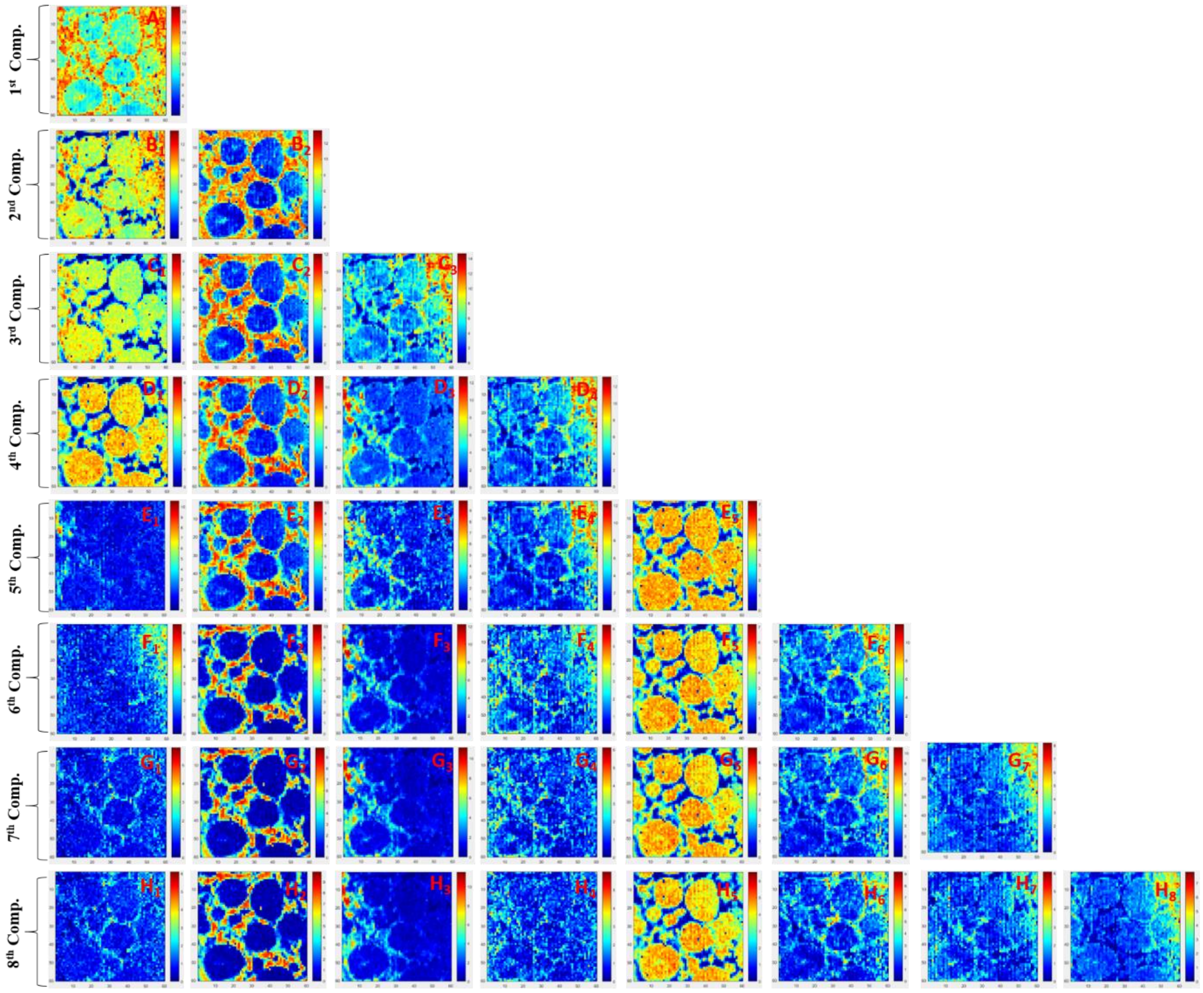

Figure S18a: Cluster-aided-MCR-ALS analysis resulted in concentration profiles (*C_{500_out}*, *3600* x *36*) and corresponding spectral profiles (*$S^t_{500\_out}$*, *36* x *1550*). (a) The concentration profiles (*C_{500_out}*, *3600* x *36*) were refolded back to form thirty-six (36) sub-pixel RCI having a dimension (C_{500_out}, *60* x *60*).

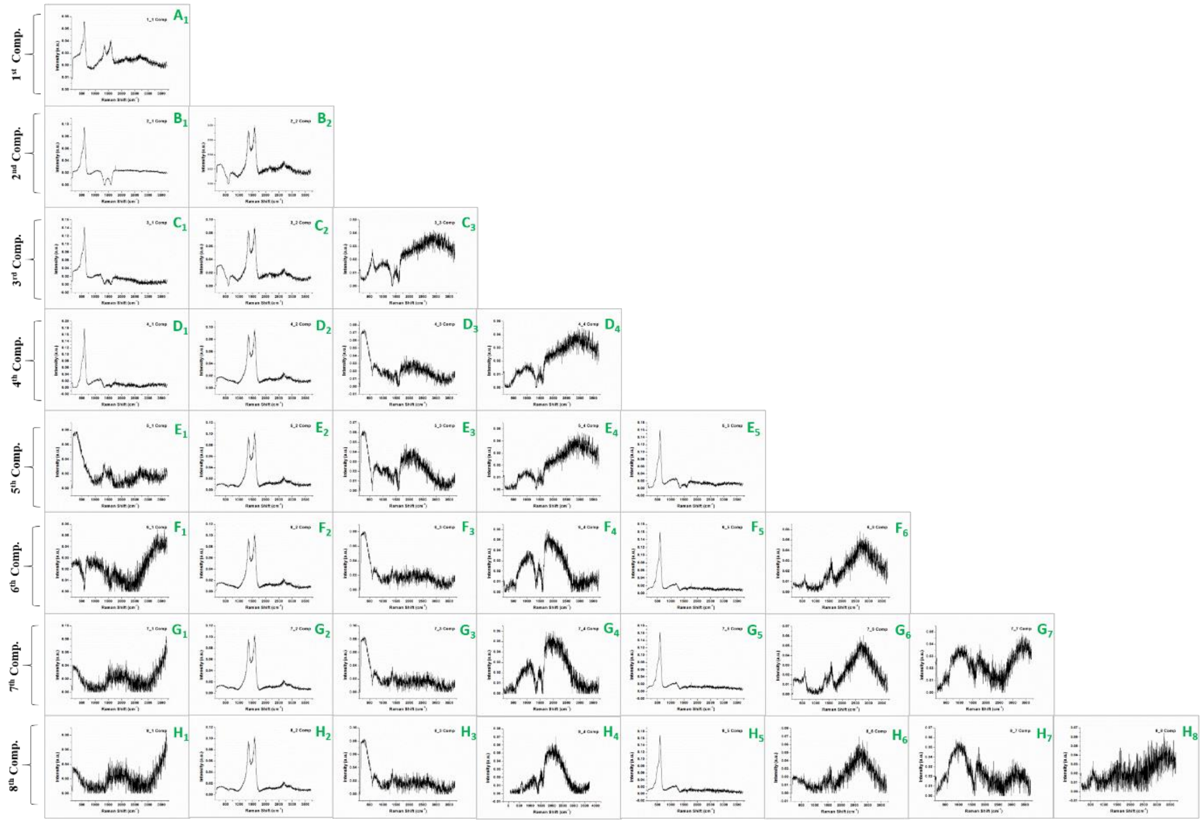

Figure S18b: Cluster-aided-MCR-ALS analysis resulted in concentration profiles (C_{500_out}, *3600* x *36*) and corresponding spectral profiles ($S^t_{500\_out}$, *36* x *1550*). spectral profiles ($S^t_{500\_out}$, *36* x *1550*).

19. Hierarchical cluster analysis (HCA) of X_{500_Out} dataset.

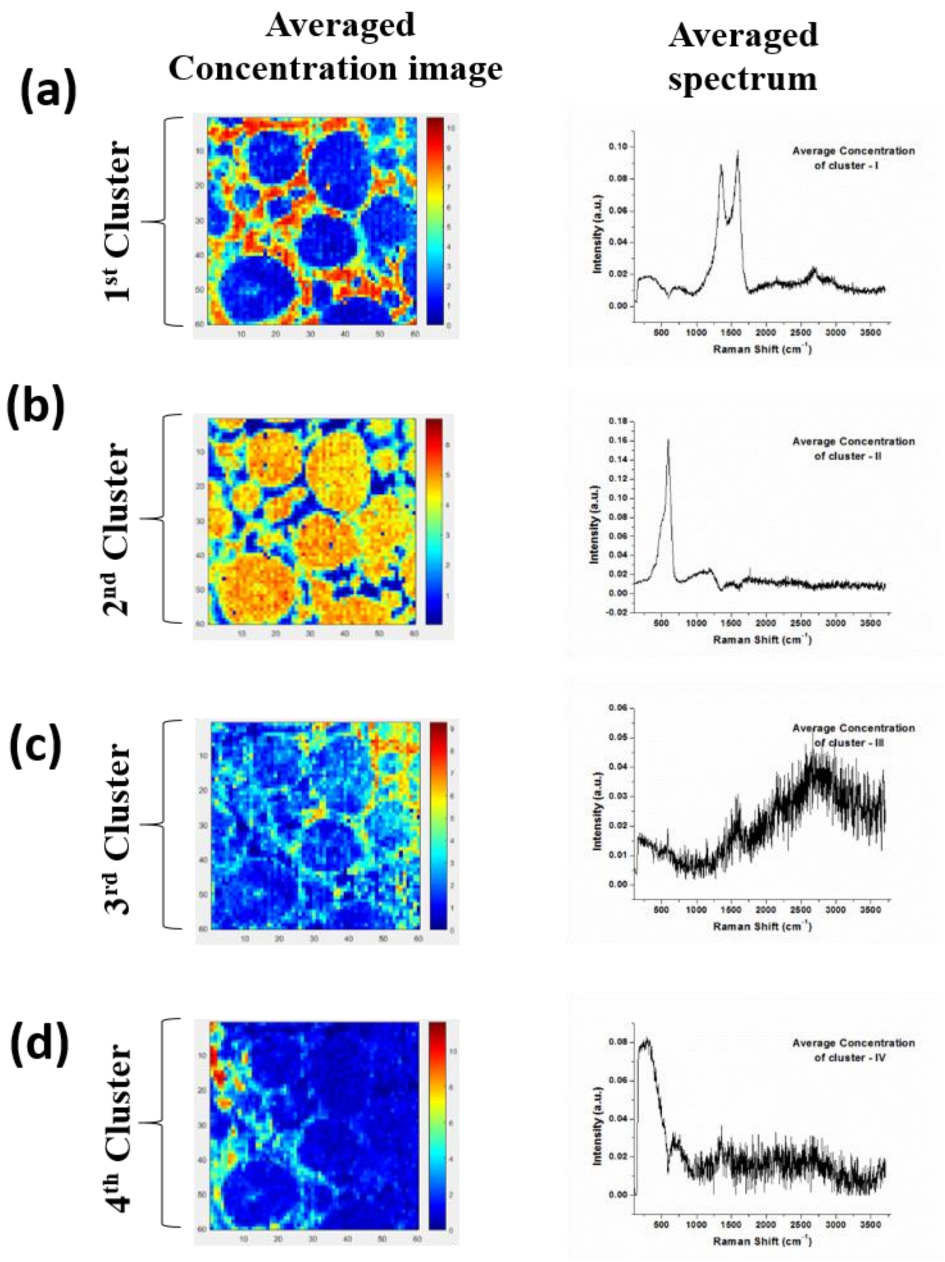

Figure S19: Hierarchical cluster analysis (HCA) of X_{500_Out} dataset. The averaged concentration image and corresponding spectra in the respective cluster can be seen.

20. Univariate vs Unsupervised intelligence (C-MCR-ALS) results of X_{500_out} dataset.

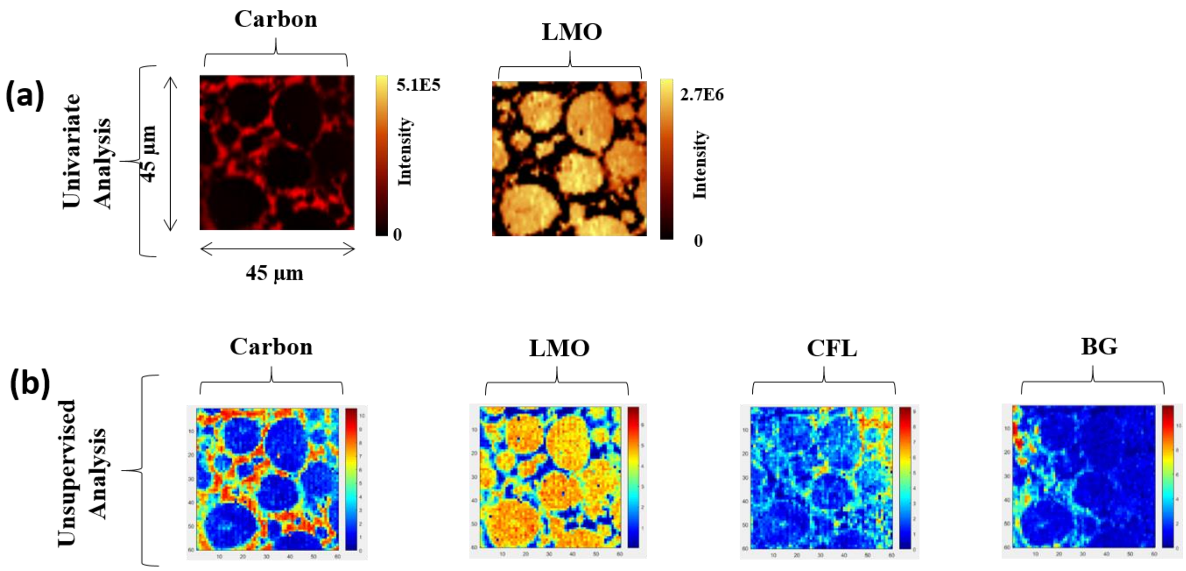

Figure S20: Univariate vs unsupervised intelligence (C-MCR-ALS) results of X_{500_out} dataset. The supervised intelligence (C-MCR-ALS) analysis depicts that carbon and LMO mapping matches exactly with univariate results.

21. In-line class labels prediction with neural network (*NN_{500_out}*) classifier.

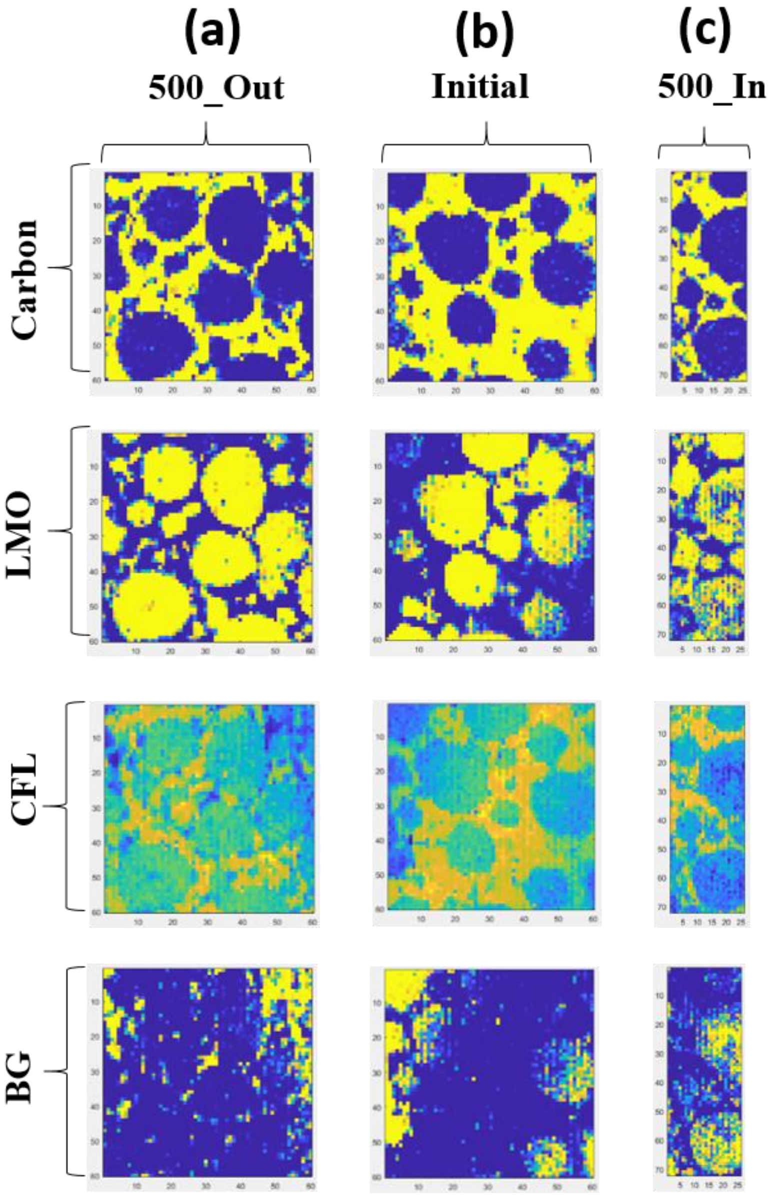

Figure S21: In-line class labels prediction with neural network (*NN_{500_out}*) classifier. Results shows the predicted concentration images for pristine, 500_Out, and 500_IN samples.

22. Silhouette-clustering on MCR-ALS extracted components and HCA clusters (pristine LIB Raman dataset).

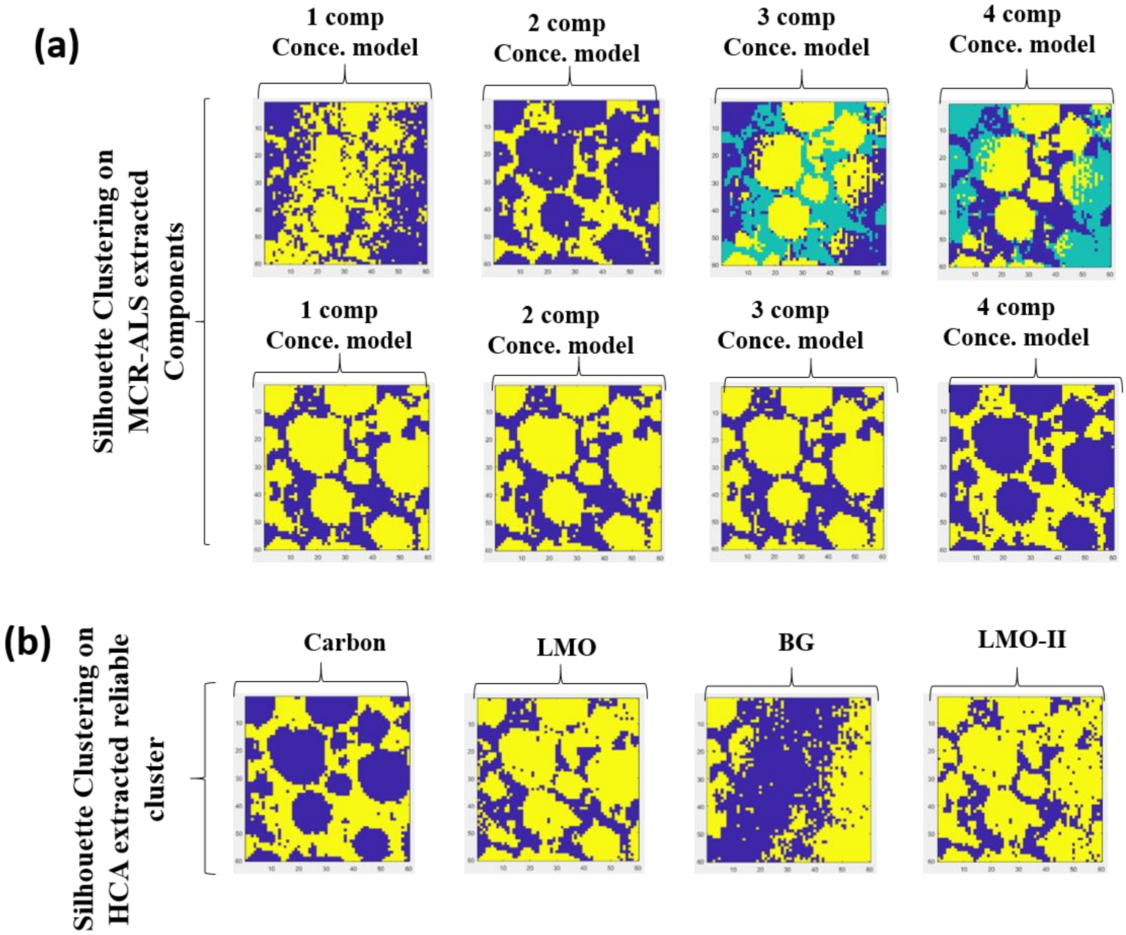

Figure S22: Silhouette-clustering on MCR-ALS extracted components and HCA clusters (pristine LIB Raman dataset). (a) Silhouette-clustering shows the presence of multiple region that is proportional to the type of component present in the dataset. (b) Silhouette-clustering on HCA clusters (pristine LIB Raman dataset) shows two distinct segmentation.

23. LMO and carbon spectrum extracted using cluster-aided-MCR-ALS analysis from pristine, 500_In, and 500_Out LIB samples.

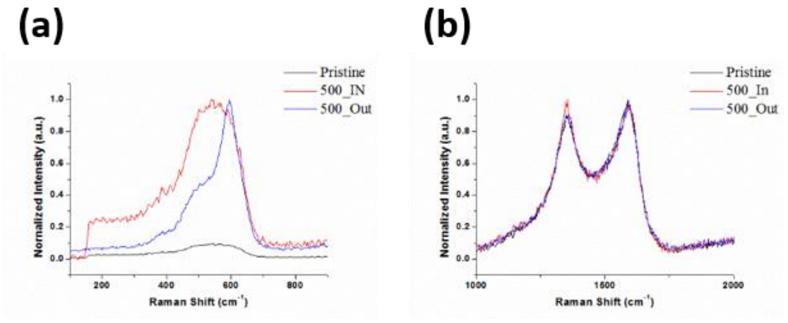

Figure S23: LMO and carbon spectrum extracted using cluster-aided-MCR-ALS analysis from pristine, 500_In, and 500_Out LIB samples.

24. LMO peak deconvolution for pristine, 500_In, and 500_Out LIB samples.

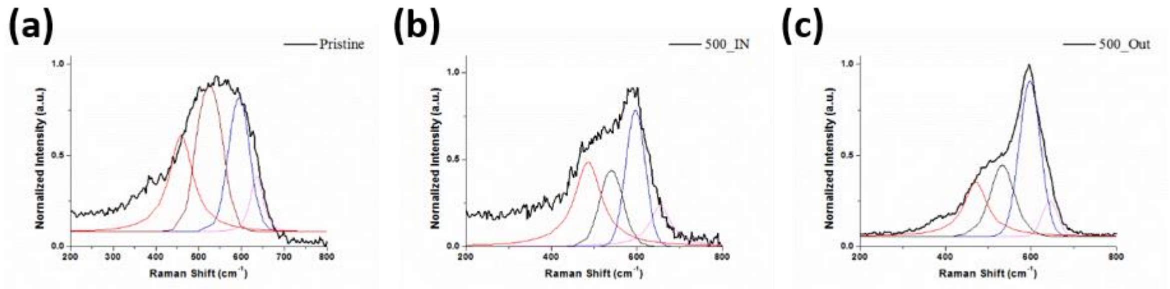

Figure S24: LMO peak deconvolution of spectrum extracted using cluster-aided-MCR-ALS. (a) Pristine, (b) 500_In, and (c) 500_Out LIB samples.

25. LMO and carbon spectrum extracted using NMF-SO-ARD from pristine, 500_In, and 500_Out LIB samples.

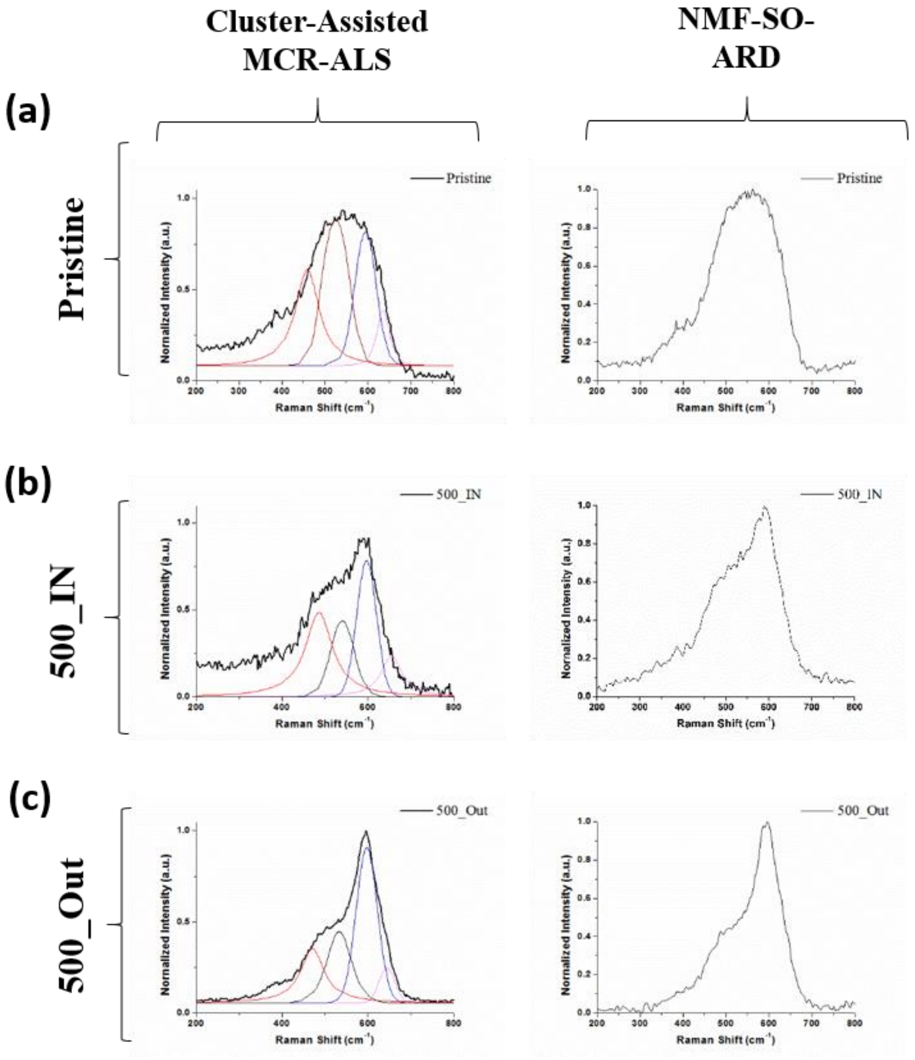

Figure S25: Comparison between the cluster assisted MCR-ALS and NMF-SO-ARD. (a) Pristine, (b) 500_In, and (c) 500_Out LIB samples.

26. Quantitative analysis of LIB electrodes.

Quantification of LMO and Carbon: Each pixel contained the concentration profile. The number of pixels with an appropriate threshold is used as a metric for quantification. We have plotted the histogram and chosen the threshold to remove the background.

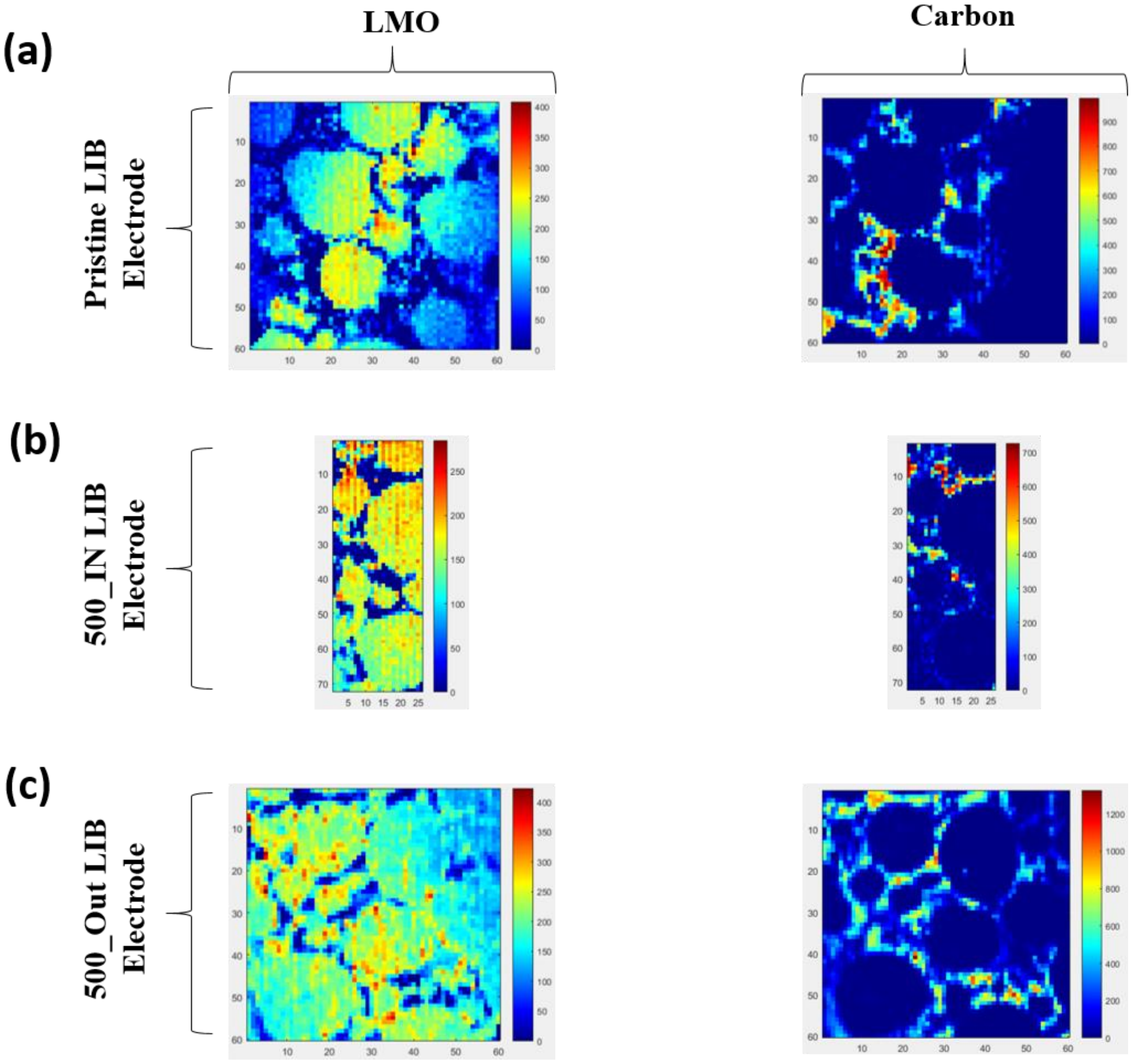

Figure S26: Quantification of LIB electrodes. (a) Pristine Electrode - LMO and carbon was 93.7 and 6.3 %, respectively. (b) 500_IN electrode - LMO and carbon was 95.2 and 4.8 %, respectively. (c) 500_Out electrode - LMO and carbon was 92.4 and 7.6 %, respectively.

27. Baseline correction model

The baseline correction model is an adaptive iteratively reweighted penalized least square algorithm. The baseline might be caused by the physiological and optical properties of the sample, along with other possible environmental and instrumental sources. The baseline should be removed prior to any analysis. The algorithm needs two parameters, namely lambda (λ) and p, respectively. The range of values for both parameters are as follows (λ: 1 - 10^8 & p: 0.001 – 1). The first parameter λ dictates how smoothly the baseline is fitted to the Raman data-set. The small-value of λ generates a baseline that closely follows the features of the natural baseline. On the contrary, the baseline becomes more linear with the higher values of λ. The value of p determines the different weights given to those points in the fitted baseline, which have positive residuals. We have fixed the p values to default 0.001 and have the first parameter λ to be varied in the valid range to remove the baseline. One can find the optimal value of λ either manually or by using the baseline regression model (BRM). However, due to the desire of higher accuracy, the optimal value of λ was estimated by the manual selection process for the pristine, 500_IN, and 500_Out LIB data-set.

Manually selection of lambda (λ): For a particular Raman data-set (ex; Pristine LIB sample: 3600 Raman spectrum), the Euclidian distance of all 3600 Raman spectrum was estimated, subsequently all the 3600 Raman spectrum was sorted based on increasing Euclidian distance. Equally spaced five Raman spectrum was selected from the sorted list and displayed in the airPLS window. As ones vary the parameter λ, the effectiveness of the baseline removal was judged visually in all five windows. The criteria of best λ is the one that effectively removes the baseline from all five windows. Once, the optimal λ was found, afterword, the airPLS algorithm was processed against the Raman data-set containing all 3600 Raman spectrum. Finally, the baseline removed data-set is stored for further analysis.

Baseline Regression Model-based selection of lambda (λ): We have various LIB Raman data-sets; it includes previously collected datasets for LIB Raman analysis done on different occasions using the same instrument (Alpha-300 confocal Raman microscope-WITec, GmbH). We have followed the similar procedure stated in the manual selection of lambda section. However, while removing the baseline after manual selection for a particular data-set, the λ was assigned as labels for the entire data-set and stored in Database (DB). After multiple such iterations, we have various Raman data-sets [data acquired in as-is foam: Data] and respective λ [labels]. Afterward, using regression analysis, a baseline regression model was easy to be trained (BRM). The efficacy of the BRM was tested for many different data-set, including the LIB data-set used in the present report. It was found that the difference between the manually selected lambda and BRM predicted lambda was found to be less than ~ 2%, in case of pristine, 500_IN, and 500_Out LIB data-set.

Table T1: Spectra acquisition information

S.No.	Sample Name	Scan Width (µm)	Scan Height (µm)	Points per line	Lines per image
1.	Pristine	45	45	60	60
2.	500_IN	55	20	72	26
3.	500_Out	45	35	60	60

Table T2: Class label information for pristine LIB electrode

S.No.	Clusters	Cluster Class Label
1.	Carbon	"C"
2.	$LiMO_2$	"LMO"
3.	Background	"BG"
4.	$LiMO_2$ + fluorescence	"LMO-II"

Table T3: Class label information for 500_IN LIB electrode

S.No.	Clusters	Cluster Class Label
1.	Carbon	"C"
2.	$LiMO_2$	"LMO"
3.	$LiMO_2$ + fluorescence	"LMO-II"
4.	Background	"BG"

Table T4: Class label information for 500_Out LIB electrode

S.No.	Clusters	Cluster Class Label
1.	Carbon	"C"
2.	$LiMO_2$	"LMO"
3.	Carbon + fluorescence	"CFL"
4.	Background	"BG"

Table T5: Raman modes for LMO phase

S.No.	Assignment of band	Band Position (cm^{-1})
1.	Ni (E_g)	470
2.	Co (E_g)	502
3.	Co (A_{1g})	524
4.	Ni (A_{1g})	551
5.	Mn (E_g)	588
6.	Mn (A_{1g})	632

Table T6: Deconvolution of LMO extracted from the pristine LIB sample.

S.No.	Band Position (cm^{-1})	Band Area	Assignment of band
1.	458.8	47	Ni (E_g)
2.	524	60	Co (A_{1g})
3.	594	45	Mn (E_g)
4.	635	12	Mn (A_{1g})

Table T7: Deconvolution of LMO extracted from the 500_IN LIB sample.

S.No.	Band Position (cm^{-1})	Band Area	Assignment of band
1.	472	18	Ni (E_g)
2.	540	45	Co (A_{1g})
3.	596	30	Mn (E_g)
4.	650	35	Mn (A_{1g})

Table T8: Deconvolution of LMO extracted from the 500_Out LIB sample.

S.No.	Band Position (cm^{-1})	Band Area	Assignment of band
1.	470	28	Ni (E_g)
2.	532	29	Co (A_{1g})
3.	597	53	Mn (E_g)
4.	644	8.8	Mn (A_{1g})
